# Motorcycle Noise Annoyance in Residential Areas Along Popular Leisure Routes †

**DOI:** 10.3390/ijerph23080966

**Published:** 2026-07-26

**Authors:** Dirk Schreckenberg, Sarah Leona Benz, Julia Kuhlmann, Jonas Bilik, Christian Popp, Frank Heidebrunn, Wolfgang Wack, Ferenc Marki

**Affiliations:** 1 ZEUS GmbH, Centre for Applied Psychology, Environmental and Social Research, D-58093 Hagen, Germany; benz@zeusgmbh.de (S.L.B.); kuhlmann@zeusgmbh.de (J.K.); bilik@zeusgmbh.de (J.B.); wolfgang.wack@mailbox.org (W.W.); 2 Lärmkontor GmbH, D-22761 Hamburg, Germanyf.heidebrunn@laermkontor.de (F.H.); 3 Department of Networked Systems and Services, Faculty of Electrical Engineering and Informatics, Budapest University of Technology and Economics, H-1111 Budapest, Hungary; marki@vik.bme.hu

**Keywords:** motorcycle noise, noise annoyance, exposure–response relationships, experience-sampling method, road traffic noise, %HA

## Abstract

**Highlights:**

**Public health relevance—How does this work relate to a public health issue?**

- Motorcycle noise along popular leisure routes represents a distinct and systematically underestimated environmental health burden: nearly half of residents living alongside heavily used motorcycle roads in Baden-Wuerttemberg (46.5%) reported high noise annoyance, a prevalence far exceeding that for any other road traffic source and substantially higher than predicted by standard road traffic exposure–response models used in European noise policy.
- Unlike general road traffic noise—distributed across all hours and seasons—motorcycle leisure traffic concentrates its acoustic impact during weekend and holiday daytime periods, precisely when residents seek relaxation. Current regulatory indicators (*L*_den_, *L*_Aeq,16h_) based on annual energy averaging fail to capture this temporally concentrated burden, leaving a significant community health impact unaddressed by existing noise action planning frameworks.

**Public health significance—Why is this work of significance to public health?**

- Source-specific exposure–response functions derived from both long-term surveys and experience-sampling data show that the 25–highly-annoyed threshold is reached at hourly motorcycle noise levels approximately 16 dB lower than for passenger car noise on weekends (≈52 dB *L*_Aeq,1h_ vs. ≈68 dB *L*_Aeq,1h_). This implies that the WHO and EU road traffic noise guideline values provide insufficient protection for residents in motorcycle-dominated settings, establishing a scientifically grounded basis for source-specific noise criteria.
- Non-acoustic factors—in particular negative attitudes towards motorcycle riders and the perceived inability to cope with motorcycle noise—are strong independent predictors of annoyance beyond the noise exposure. This finding, consistent across two independent European studies (Baden-Wuerttemberg and the Austrian Alps), demonstrates that community health protection in motorcycle-noise contexts requires strategies that address both the physical noise signal and the social dimensions of source perception.

**Public health implications—What are the key implications or messages for practitioners, policy makers and/or researchers in public health?**

- Local noise action planning under the Environmental Noise Directive should incorporate source-specific assessment for routes with significant motorcycle leisure traffic, using hourly noise metrics (on weekends: *L*_Aeq,1h_ ≤ 52 dB; *L*_AFmax,1h_ ≤ 78 dB; N_60_ ≤ 25 motorcycle events per hour) as operationally applicable thresholds; these values, converging with findings from the Austrian Alpine study, provide practitioners with evidence-based criteria that are substantially more protective than generic road traffic standards.
- Effective noise management along motorcycle leisure corridors requires a dual strategy: technical emission-reduction measures (stricter type-approval noise limits, speed management on sensitive routes, incentives for quieter motorcycles) must be combined with communication and participatory governance approaches that engage both the motorcycling community and affected residents, addressing the attitudinal and social dimensions of annoyance that purely acoustic interventions leave unresolved.

**Abstract:**

Motorcycle noise along leisure routes constitutes a distinct environmental health burden poorly captured by standard road traffic noise indicators. This study derives source-specific exposure–response functions and examines non-acoustic predictors of residential motorcycle noise annoyance. A mixed-methods socio-acoustic design was applied in five study areas in Baden-Wuerttemberg, Germany. A community survey (*N* = 493) assessed long-term annoyance (12-month recall); a smartphone-based experience-sampling study (MotoApp; *N* = 213; ten days in summer 2022) collected hourly ratings. Acoustic measurements provided vehicle-specific *L*_Aeq,1h_, *L*_AFmax,1h_, and N_60_; exposure–response functions were estimated using logistic regression and generalised estimating equations. In the long-term survey, 46.5% were highly annoyed by motorcycle noise, compared with 18.4% for passenger cars. Motorcycle exposure–response curves were markedly shifted; the 25–highly-annoyed threshold was reached approximately 16 dB lower in *L*_Aeq,1h_ than for passenger cars on weekends. Negative attitudes towards motorcycle riders, low coping capacity, and noise sensitivity were significant independent predictors. Source-specific assessment is necessary for motorcycle leisure routes. The 25–highly-annoyed criterion maps to approximately 52 dB *L*_Aeq,1h_ and 78 dB *L*_AFmax,1h_ on weekends, and to 25 N_60_ events per hour—substantially below current road traffic guideline values—providing actionable thresholds for noise action planning.

## 1. Introduction

### 1.1. Environmental Noise as a Public Health Challenge

Environmental noise has been identified as the second-most harmful environmental stressor in Europe after air pollution, contributing substantially to cardiovascular disease, high sleep disturbance, cognitive impairment in children, and high noise annoyance [1]. The European Environment Agency [2] estimates 1.2 M healthy life years lost (DALYs, disability-adjusted life years) due to road traffic noise alone in the European Union in areas covered by the environmental noise mapping according to the European Noise Directive (END) 2002/49/EC [3] (*L*_den_ ≥ 55 dB; *L*_night_ ≥ 50 dB) and 1.4 M DALYs in areas covered by the END with exposure above WHO recommendations (*L*_den_ ≥ 53 dB; *L*_night_ ≥ 45 dB for road traffic noise). Road traffic constitutes the dominant source of environmental noise exposure for European urban and peri-urban populations, accounting for the majority of highly annoyed and highly sleep-disturbed residents [1,2,3,4,5,6]. In response, the END [3] established harmonised noise indicators—*L*_den_ and *L*_night_—and requires member states to produce strategic noise maps and action plans for major agglomerations and transport infrastructure.

Despite this regulatory framework, the growing body of socio-acoustic evidence indicates that generic exposure–response relationships derived from mixed-traffic streams do not uniformly represent community responses to noise from all vehicle types. In particular, motorcycles—whose contribution to total traffic volume is comparatively modest—have been increasingly recognised as a source of disproportionate annoyance and sleep disturbance relative to their energy contribution to the broadband noise level [7,8,9]. The present study adds to the body of literature on this gap.

### 1.2. Characteristics of Motorcycle Noise and Its Specific Annoyance Potential

Motorcycles are characterised by high instantaneous emission levels, pronounced tonal and impulsive components, and a distinctive spectral signature shaped by engine and exhaust harmonics [10]. These characteristics are not fully captured by A-weighted equivalent continuous sound pressure level-type metrics, the primary metrics used in regulatory noise assessment. Psychoacoustic analyses have demonstrated that annoyance responses to motorcycle noise correlate strongly with perceptual attributes, including loudness, sharpness, roughness, and fluctuation strength, in addition to overall sound pressure level, implying that the A-weighted equivalent sound pressure level common to standard assessment methods systematically underestimates the perceptual salience of motorcycle noise events [10,11,12,13].

A further distinctive feature of motorcycle noise in leisure contexts is its pronounced temporal structure: leisure riding is concentrated on warm, dry summer weekends and public holidays, producing weekend-peaked acoustic conditions that differ from the temporally distributed noise climate on weekdays, for which standard exposure indicators and annoyance models were derived [7,8]. Residents in communities adjacent to popular scenic routes are consequently exposed to a noise environment characterised by high instantaneous levels, frequent impulsive events, and seasonal concentration—all features likely to elevate annoyance beyond what annual energy averages would predict [8].

A further dimension of motorcycle noise annoyance concerns its psychological framing. Unlike background traffic noise, which residents may habituate to over time, motorcycle noise is frequently attributed to specific, identifiable human activities—specifically to perceived rider behaviour (high engine revs, speed, risk-taking). This attitudinal dimension, as well as attitudes in general related to the noise source and authorities regarded as responsible for the noise and its mitigation, have been identified as robust non-acoustic predictors of community annoyance in several studies [7,8,14,15].

### 1.3. State of Research on Motorcycle Noise and Exposure–Response Relationships

Quantitative socio-acoustic studies specifically examining motorcycle noise annoyance remain relatively sparse compared with the extensive literature on (generic) road traffic, aircraft, and railway noise. The work of Nelson [16] established that motorcycle noise is particularly salient in low-background acoustic environments, where individual prominent events dominate the soundscape. More recent psychoacoustic experiments by Samani and Altinsoy [10] confirmed that spectral content—particularly sharpness and specific tonal energy—accounts for variance in annoyance independently of overall sound pressure level.

One of the most methodologically robust community-level exposure–response data for motorcycle noise currently available originates from a cross-sectional study conducted by Lechner and colleagues along motorcycle routes in the Alpine valleys in Austria [7]. The study derived source-specific logistic exposure–response functions and found that the %HA curve for motorcycle noise was shifted by more than 30 dB to lower levels relative to standard road traffic curves—meaning that a given percentage of highly annoyed residents was reached at substantially lower motorcycle-specific sound levels than the generic road traffic noise annoyance model [4] would predict. This leads the authors to conclude that a 5 dB malus for motorcycle noise, as suggested by other authors [11,17], is far too low.

Annoyance in the Alpine study was concentrated on weekend days. According to the authors, it was largely independent of the background exposure from other road traffic, suggesting that residents cognitively and perceptually disentangle motorcycle noise from the general traffic background.

Two conference papers from the ICBEN 2023 Congress in Belgrade reported interim findings from the Baden-Wuerttemberg Motorcycle Noise Study, which forms the empirical basis of the present article [8,18]. Part I [8] documented long-term community annoyance responses among 493 residents living alongside busy motorcycle routes, reporting a prevalence of highly annoyed individuals of 46.5% and significant correlations between annoyance and attitudinal variables. Part II [18] reported short-term annoyance dynamics during the day and across weekdays and weekends from an experience-sampling component, confirming source-specific exposure–response differences across vehicle classes and identifying weekday–weekend asymmetries.

Contextual evidence from other geographic settings corroborates the specific potential of annoyance from motorcycle-rich traffic streams. In residential areas of Makassar, Indonesia, equivalent continuous levels (*L*_Aeq_) between 62 dB (with a motorcycle proportion of 25–50%) and 85 dB (with a motorcycle proportion of 75–100%) were documented on streets with high motorcycle proportions, exceeding local guideline values (73 dB) [19]. At the level of regulatory framing, Dendale and Münzel [20] have recently argued that motorcycles deserve substantially greater attention within European environmental noise and cardiovascular health policy, noting the absence of motorcycle-specific limit values in the Environmental Noise Directive.

Despite this emerging evidence base, key gaps remain. No published study has combined long-term community surveys with short-term experience-sampling methodology (ESM) to characterise both chronic and acute annoyance dynamics in a motorcycle-noise context. There are currently no German-specific formal exposure–response functions for motorcycle noise as a distinct source. The relative contributions of acoustic and non-acoustic factors—including attitudes and noise sensitivity—to noise annoyance remain incompletely quantified. Finally, no evidence-based, source-specific noise assessment criteria have been proposed for application to motorcycle-dominated leisure routes in the German or European regulatory context.

### 1.4. Research Objectives and Hypotheses

The present study addresses these gaps through a mixed-methods socio-acoustic field investigation conducted in Baden-Wuerttemberg, southwest Germany. The study pursued three primary research objectives:

**RQ1.**  Are source-specific exposure–response curves (%HA) for motorcycle noise, derived from both retrospective surveys on long-term noise annoyance and hourly ESM assessments, on short-term annoyance shifted relative to %HA curves for noise from other vehicles?

**RQ2.**  Is the exposure–annoyance relationship for motorcycles stronger on weekends than on weekdays, independently of the noise level itself?

**RQ3.**  Do non-acoustic individual-level factors—specifically noise sensitivity, attitudes towards motorcycles, and coping capacity—moderate the exposure–annoyance relationship?

Based on prior literature, the following hypotheses are formulated:

**H1.**  *Motorcycle noise produces higher annoyance probabilities than cars or lorries at equivalent* *L*_Aeq_ *values.*

**H2.**  *The weekday–weekend differential is more pronounced for motorcycles than for other vehicle classes.*

**H3.**  *Negative attitudinal appraisal of motorcycle riders is an important non-acoustic predictor after controlling for noise exposure).*

This article is a revised and expanded version of two conference papers published in the Proceedings of the 14th ICBEN Congress on Noise as a Public Health Problem in Belgrade, Serbia, 18–22 June 2023 [8,18].

## 2. Materials and Methods

### 2.1. Study Design

This investigation employed a mixed-methods socio-acoustic design combining (a) a cross-sectional, population-based postal and online community survey (long-term component) with (b) an experience-sampling study [21] with a subsample of (a) using a purpose-built smartphone application (the so-called ‘MotoApp’ short-term component). Both components were deployed during summer 2022, enabling the analysis of long-term noise annoyance (12-month retrospective recall) and the assessment of hourly noise annoyance several times per day over 10 consecutive days.

Participation was entirely voluntary. All participants were fully informed before enrolment about the purpose of the study, the nature of the data to be collected, the identity of the responsible research institution, and their rights under applicable data protection law. For the long-term survey, participants’ informed consent was documented by returning the completed questionnaire or submitting the online form, following a written participant information sheet included with the invitation. For the ESM component, participants provided explicit digital consent within the MotoApp before activating the diary function.

All data were collected, stored, and processed in strict accordance with the General Data Protection Regulation (EU) 2016/679 (GDPR) and applicable national data protection legislation. No personally identifiable data are reported in this article; all analyses are based on anonymised datasets.

This study did not require approval by an Institutional Review Board or ethics committee. Both study components—the postal/online community survey and the smartphone-based experience-sampling study—were purely observational in design and involved the collection of self-reported questionnaire data exclusively. The exemption from formal ethics review is consistent with the legal and institutional framework applicable to non-interventional social science survey research of this type in Germany, in which ethical approval is not mandated for anonymous or anonymised surveys conducted with fully informed adult participants where no sensitive personal data within the meaning of Article 9 GDPR are processed. There is no risk of harm to participants [22].

### 2.2. Study Areas

**Figure 1:**
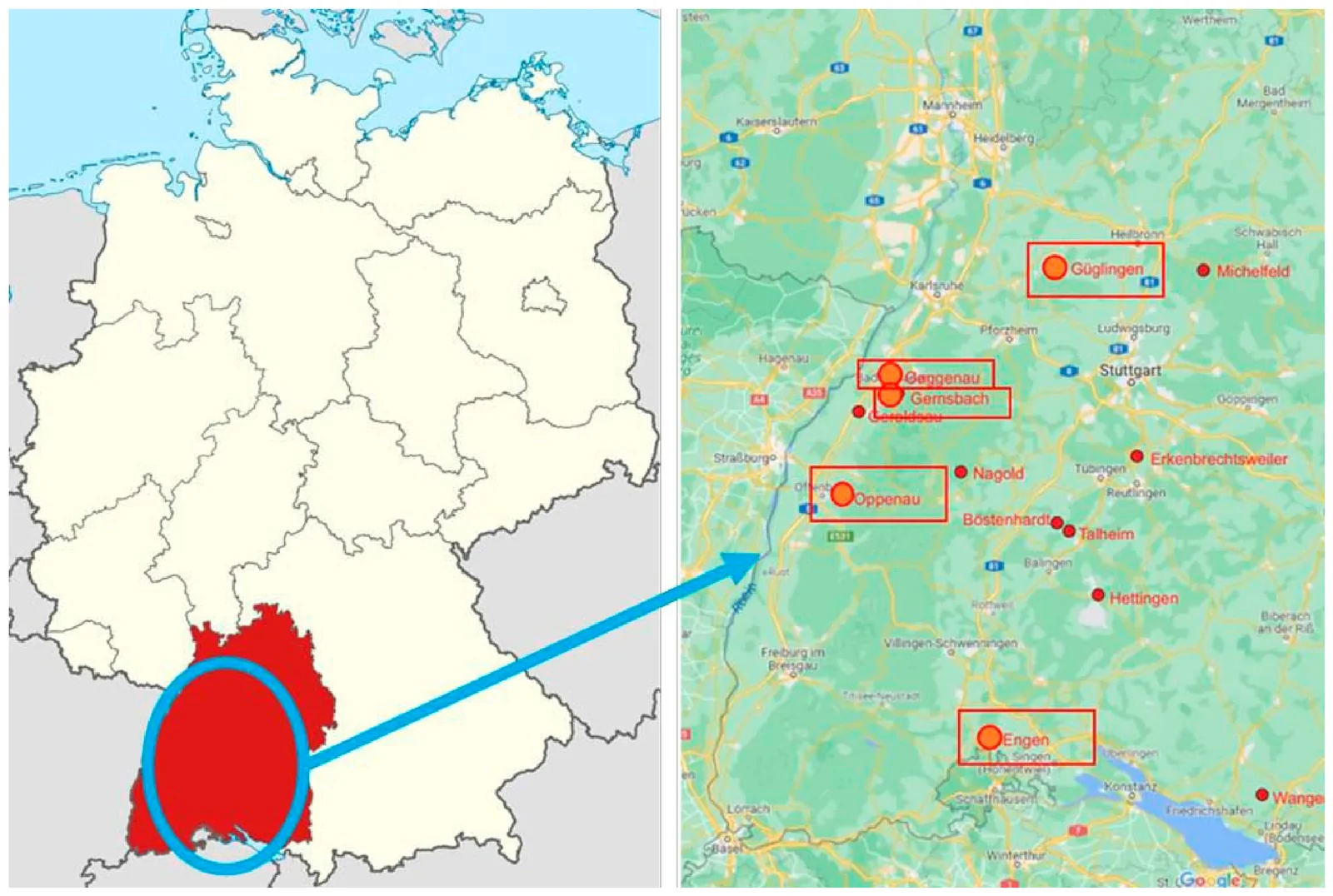
Study areas of the motorcycle noise study in Baden-Wuerttemberg, Germany [8].

Five study areas were selected along heavily used motorcycle leisure routes in Baden-Wuerttemberg, southwest Germany: (1) Engen in the Hegau region; (2) Oppenau in the northern Black Forest; (3) Gernsbach in the Murgtal valley; (4) Gaggenau-Michelbach; and (5) Güglingen in the Zabergäu region (Figure 1). Selection criteria included documented high motorcycle traffic volumes during summer weekends, primarily residential land use adjacent to the route, topographic variation (enabling assessment of propagation effects), and logistical accessibility for continuous acoustic monitoring.

All five areas are characterised by scenic, curvilinear road alignments through forested hills or river valleys that attract leisure motorcyclists, particularly on Saturdays, Sundays, and public holidays between April and October. Table 1 shows the (annual) average hourly road traffic volume along the motorcycle routes in the study areas based on traffic census data of 2020 and 2021. Except for Oppenau, the percentage of motorcycles was below 10% overall and below 15% on weekends. On the motorcycle route in Oppenau, the overall share of motorcycles was 27%, rising to 38% on weekends.

During the measurement period, weekday traffic in these areas is dominated by private cars, with the proportion of lorries generally low. Weekend motorcycle traffic volumes ranged from 1 to 138 vehicles per hour (average: 52 vehicles per hour) at the monitoring points, accounting for about 10% of total traffic flow during high-volume hours. On weekdays, the motorcycle traffic volume ranged from 1 to 74 vehicles (average 24 vehicles) per hour (4.5% of total traffic volume during high-volume hours). The areas are rural, characterised by relatively low industrial and commercial activity and higher agricultural activity.

### 2.3. Long-Term Community Survey

#### 2.3.1. Sampling and Data Collection

An address-based random sample of adult residents aged 18 years and older was drawn from registration data of the municipal registration offices of the five study areas. Sampled individuals received a postal invitation to complete either a paper questionnaire or an equivalent online survey (parallel design). The invitation letter included invitations to both study parts, information about the study’s aim, the contracting authority (Ministry of Transport Baden-Wuerttemberg, Germany), the study team, and data protection in accordance with the European General Data Protection Regulation (GDPR), the link to the online questionnaire and, for optional use, the paper-pencil questionnaire, and an additional flyer with the summary of the study. The long-term survey period ran from May to June 2022.

In total, 2500 individuals were invited, of whom 493 filled in the questionnaire (response rate: 19.7%). A total of 293 individuals (59.4%) completed the paper-pencil questionnaire, and 200 completed the online version (40.6%).

#### 2.3.2. Questionnaire

Noise annoyance was assessed using the standardised verbal rating scale specified in ISO/TS 15666:2021 [23]. Respondents were asked to indicate, on a verbal five-point rating scale including the categories ‘not at all’, ‘slightly’, ‘moderately’, ‘very’, and ‘extremely’, how much they were annoyed, disturbed, or bothered by noise from motorcycles, passenger cars, lorries, coaches, and agricultural traffic over the past 12 months. The proportion of respondents indicating ‘very’ or ‘extremely’ was operationalised as the percentage highly annoyed (%HA_V_), consistent with international convention [23]. The subscribed letter “V” indicates that the 5-point rating scale is verbal.

Activity disturbance was assessed with items enquiring about interference with outdoor relaxation, sleep, communication, and concentration during the past 12 months. Residential satisfaction was measured using separate five-point items addressing satisfaction with the surrounding neighbourhood and with the dwelling itself.

The perceived capacity to cope with motorcycle noise was measured with six items (*α* = 0.85) using a 5-point agreement rating scale from (1) ‘not agree’ to (5) ‘agree very’.

Noise sensitivity was measured by a self-report item on a five-point scale from ‘not sensitive’ to ‘very sensitive’, consistent with the single-item approach used in several large-scale community noise surveys [7,8].

Attitudes towards motorcycle riding were assessed using two multi-item subscales from Lechner et al. [7]: a positive attitude dimension (capturing associations with freedom, nature experience, and recreational value; *α* = 0.75) and a negative attitude dimension (capturing perceptions of recklessness, danger, and disrespectfulness; *α* = 0.81).

Socio-demographic variables collected included age, sex, house ownership, dwelling type, years of residence at the current address, and socio-economic status (SES), self-reported on a 7-point scale visually presented as a ladder (stave 1 = low SES, stave 7 = high SES) with participants being asked to consider education, occupation and income for their SES assessment.

### 2.4. Short-Term Experience-Sampling Study (MotoApp Component)

#### 2.4.1. Participants and Recruitment

Survey respondents who indicated willingness to participate in the MotoApp component during the first study part were invited to download the ‘MotoApp’ (for Android or iOS devices) and complete the smartphone-based diary during the MotoApp field period of 2 × 5 consecutive days within a 14-day period in July 2022. These participants received written instructions explaining how to install the MotoApp and when and how to use it. Of the 493 long-term survey respondents, 213 participants downloaded the application. They contributed to the study by completing a total of 4040 short questionnaires at home, either inside or outside their dwellings. MotoApp participants were compared with non-participants on key survey variables. MotoApp participants were on average six years younger than non-participants (t(447.83) = 7.680, *p* < 0.01). Although MotoApp participants were more exposed to road traffic noise (*L*_den_) than non-participants (t(463.95) = −2.638, *p* < 0.01), no statistically significant differences between these two groups were observed in long-term annoyance due to motorcycle and other vehicles’ noise in *t*-test comparisons and in ANCOVAs when controlled for noise exposure (*L*_den_) (*F*_road_(1.482) = 0.810, *p* = 0.369; *F*_motorcyle_(1.484) = 1.428, *p* = 0.233; F_car_(1.475) = 0.525, *p* = 0.469). MotoApp participants and non-participants also did not differ in noise sensitivity (t(470.86) = 0.627, *p* = 0.531), indicating minimal selective participation bias in the primary outcome variables and personal characteristics.

#### 2.4.2. Signalised Interval-Contingent MotoApp Protocol

An interval-contingent ESM design [24] was applied for the MotoApp assessments. Table 2 illustrates the MotoApp study design and assessment times. The MotoApp delivered assessment prompts at six 2 h intervals per day, spanning the core monitoring period from 8 a.m. to 8 p.m. One group did the assessments in the first 5-day period at 9 a.m., 11 a.m., 1 p.m., 3 p.m., 5 p.m., 7 p.m., and in the second 5-day period at 10 a.m., 12 p.m., 2 p.m., 4 p.m., 6 p.m., 8 p.m. The other group started at 10 a.m. in the first 5-day period. It continued at 9 a.m. in the second 5-day period, followed by five further assessments per day at 2 h intervals. Participants were randomly distributed to one of these two groups. The first 5-day period was from Wednesday to Sunday, the other one started a week later, from Friday to Tuesday. The participants of the MotoApp study received an incentive of 70 € for their participation.

Participants were encouraged to respond immediately after receiving the notification, allowing a 15 min window for responses.

At each prompt, participants indicated their location (at home, indoor/outdoor, away). If at home, inside, the window position (closed, tilted, open) was rated as well as the annoyance due to noise from motorcycles, passenger cars, and lorries within the past hour, using the five-point ISO/TS 15666-verbal scale.

### 2.5. Noise Exposure Assessments

#### 2.5.1. Noise Exposure Modelling for the Long-Term Survey

For the long-term exposure–response analysis, annual average road traffic noise indicators (*L*_den_ and daytime *L*_Aeq,16h_ for 6 a.m.–10 p.m.), *L*_night_ for 10 p.m.–6 a.m.) were computed at the highest-exposed (‘loudest’) façade of each survey respondent’s residence using the German road traffic noise modelling procedure RLS-19 [25]. Calculations were performed using the SoundPLAN 8.2–64-bit software, version of 19 September 2022. For this article, results referring to *L*_den_ are presented.

Traffic volume data for the roads included in the study as model inputs were obtained from the Ministry of Transport Baden-Wuerttemberg’s Traffic Monitoring 2018. For this study, vehicle-specific long-term noise exposure metrics were not available. Thus, we used the total road traffic noise exposure indicators for the exposure–response analysis on long-term annoyance.

#### 2.5.2. Acoustic Measurements During the MotoApp Field Phase

During the MotoApp field phase, continuous acoustic monitoring was conducted at twelve fixed measurement points distributed across the five study areas along the motorcycle routes. Measurements were done using the reflector post radar measurement device TOPO.SLP 46 manufactured by RTB GmbH & Co. KG, Bad Lippspringe, Germany. The device allows vehicle-specific noise measurements in terms of the pass-by sound pressure level (SPL) via a built-in microphone, combined with certified vehicle classification using radar measurements of speed, vehicle length, and the number and configuration of vehicle axles. The SPLs for each vehicle were summed to obtain vehicle-specific and total (all vehicles) SPLs per hour, from which the following metrics were estimated.

At each monitoring point, the following acoustic parameters were derived for each hour of the measurement period (8 a.m. to 8 p.m.):

- The vehicle-class-specific equivalent continuous sound pressure level (*L*_Aeq,1h_) for motorcycles, passenger cars, and lorries, calculated from the SPL per hour averaged over the hour.
- The maximum A-weighted sound pressure level of all events per hour in an hour per vehicle class (*L*_AFmax,1h_), i.e., it is the maximum sound levels of all vehicle-specific pass-bys within one hour measured with fast time weighting of 125 ms.
- Related to the *L*_AFmax,1h_, the number of events exceeding an A-weighted maximum sound level (*L*_AFmax_) 60 dB per hour per vehicle class (number above threshold of *L*_AFmax_ = 60 dB, N_60_).

Through sound propagation modelling in accordance with the modelling procedure RLS-19 [25], noise levels at the “loudest” façade were estimated again using the SoundPLAN 8.2–64 bit software, version of 19 September 2022.

### 2.6. Statistical Analysis

All analyses were conducted in the statistical software R, Version 4.2.1 [26] using the packages ggplot2 [27], dplyr [28], and geepack [29]. Significance was assessed at α = 0.05 (two-tailed).

Exposure–response modelling (long-term): Binary logistic regression was used to model the probability of HA_V_ for motorcycles, cars, and lorries separately, as a function of *L*_den_, for road traffic noise. Predicted %HA_V_ curves were plotted over the full exposure range with 95% confidence intervals.

Exposure–response modelling (short-term, MotoApp): Multilevel logistic regression was employed to account for the nested structure of the MotoApp data (repeated assessments within persons; persons within study areas) using the generalised estimating equations (GEE) approach. Fixed effects included hourly *L*_Aeq,1h_ per vehicle class (Level 1) and attitude and sensitivity scores (Level 2). Random intercepts were specified at the person level; models with random slopes for the exposure–annoyance relationship were estimated and compared using likelihood ratio tests. Separate models were estimated for weekdays and weekends. To examine hourly N-based exposure–response relationships, parallel models were estimated with N_60_ and N_70_ per hour as the exposure predictors. In the same way, exposure–response relationships were estimated for the hourly maximum sound level *L*_AFmax,1h_.

The following AI tools supported the manuscript preparation: NotebookLM by Google [30] was used for summarising the technical reports of the study, Perplexity by Perplexity AI [31] was used for updating the search for additional literature, and Claude by Anthropic [32] was used for generating the paper structure and editing certain paragraphs.

## 3. Results

### 3.1. Sample Characteristics and Acoustic Exposure Distribution

The long-term survey sample comprised 493 respondents (mean age = 52.7 years, *SD* = 17.2; 48.7% female; 75.9% homeowners; mean years of residence 19.6 years, *SD* = 16.7). A total of 213 of the long-term survey respondents participated in the MotoApp survey (mean age = 46.3 years, *SD* = 16.3; 47.4% female; 69.0% owner-occupiers; mean years of residence 16.3 years, *SD* = 15.1). Descriptive statistics for the full sample and the MotoApp subsample are presented in Table 3.

*Long-term study:* Noise exposure modelled for the highest-exposed (‘loudest’) façade at the home of the long-term survey respondents ranged from 37 to 74 dB *L*_den_ (*M* = 53.8, *SD* = 7.8). For the long-term survey, vehicle-specific noise exposure data were not available. Long-term annoyance was highest for motorcycle noise compared with passenger car and lorry noise, in both the total sample and the participants of the MotoApp study.

*MotoApp study:* Motorcycle-specific hourly equivalent levels (*L*_Aeq,1h_) during the monitoring period in the MotoApp study ranged from 11 to 67.5 dB on weekdays and from 20 to 73 dB on weekends, with weekend *L*_Aeq,1h_ values exceeding weekday *L*_Aeq,1h_ values by 4 dB on average. With about 50 dB *L*_Aeq,1h_, the mean *L*_Aeq,1h_ is higher for passenger car noise than for motorcycle and lorry noise, both on weekdays and weekends. The *L*_Aeq,1h_ for lorry noise is lower on weekends than on weekdays due to Sunday traffic restrictions for lorries and, thus, a considerably reduced hourly number of lorries at weekends. The mean hourly annoyance value due to motorcycle noise is higher on weekends than on weekdays (from Monday to Friday); for passenger car noise the average annoyance on weekends and weekdays is almost the same and for lorry noise the average noise annoyance value is lower on weekends than on weekdays. All in all, mean hourly annoyance values are considerably lower than the average long-term annoyance judgements.

Whereas the N_60_ events for passenger cars and lorries decreased on weekends, the number of N_60_ events for motorcycle traffic increased on weekends compared to weekdays. The noise exposure metrics for the long-term study and the MotoApp study are presented in Table 4.

### 3.2. Prevalence of High Annoyance

In the long-term survey, 46.5% of respondents were classified as highly annoyed (%HA_V_) by motorcycle noise, compared with 18.4% for passenger cars, 19.3% for lorries, 6.2% for coaches, and 8.7% for agricultural traffic. Overall, 25.1% reported high annoyance from road traffic noise. Thus, motorcycle noise was identified as the most annoying source by the largest proportion of respondents in all five study areas. Source-specific annoyance prevalences are summarised in Table 5, taken from [8].

### 3.3. Exposure–Response Relationships for High Noise Annoyance

#### 3.3.1. Long-Term Exposure–Response Curves

**Figure 2:**
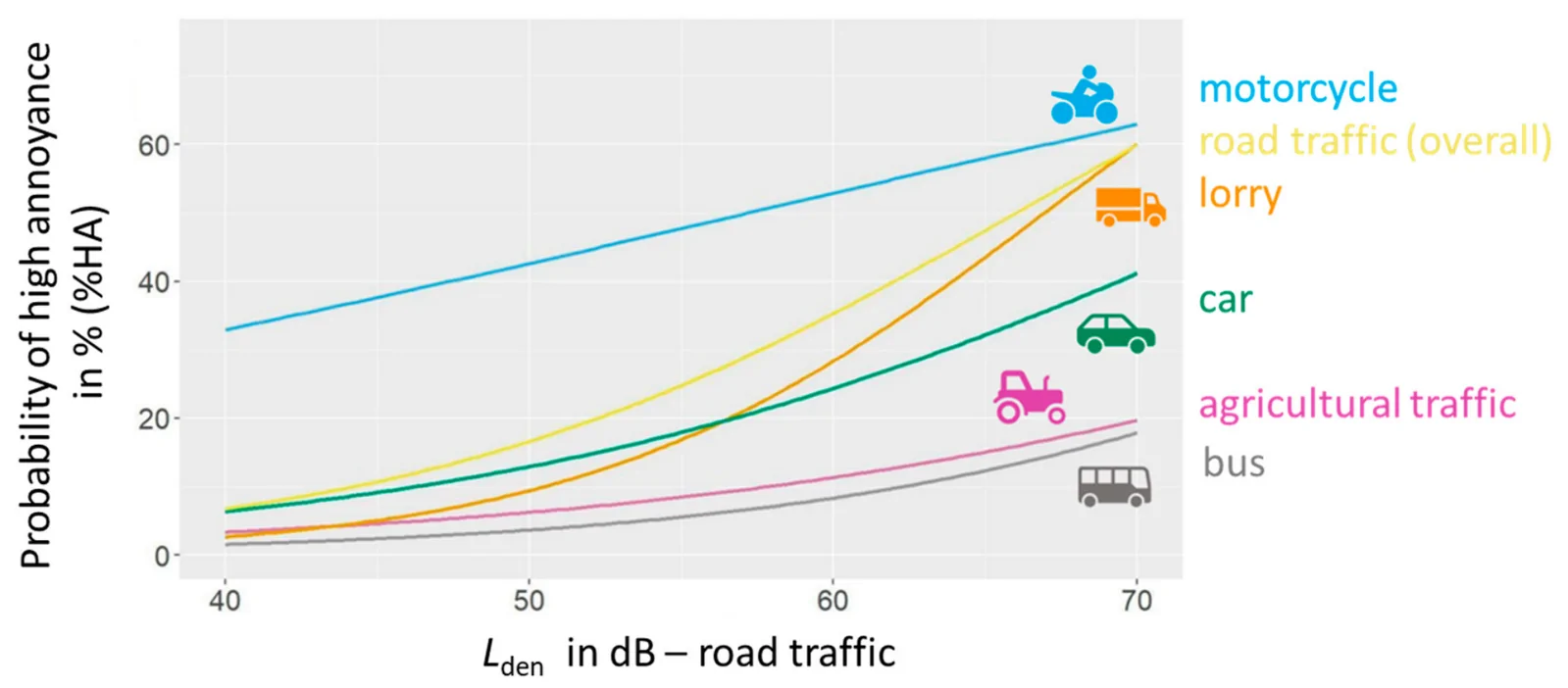
Exposure–response curves for %HA_V_ against *L*_den_ for road traffic noise in dB for noise from several vehicles of road traffic based on logistic regressions [8].

Logistic exposure–response modelling of long-term %HA_V_ as a function of modelled *L*_den_ yielded source-specific curves for motorcycles, passenger cars, and lorries. The motorcycle %HA_V_ curve lies substantially above the %HA_V_ curves of the other vehicles. For example, at equivalent *L*_den_ values, the predicted probability of high annoyance from motorcycle noise was about 20% higher than that from passenger car noise and even higher than that from agricultural traffic and coaches. The probability of high annoyance due to lorry noise was approximately 25% points lower than for motorcycle noise below 55 dB *L*_den_. Above 55 dB *L*_den_, the %HA_V_ curve for lorry noise converges with the %HA_V_ curve for motorcycle noise. The exposure–response curves are presented in Figure 2 (see also [8]), and the regression coefficients are shown in Table 6.

Several aspects contributed to motorcycle noise annoyance, including characteristics of the motorcycle sound, time of day, week, and season, the activities disturbed by the noise, and personal and social factors such as attitudes towards motorcycles and motorcyclists and noise sensitivity (see also [8]).

Results presented in Table 7 (see also [8]) indicate that motorcycle noise annoyance is higher in the afternoon and evening, on Sundays or holidays in warmer seasons (summers, spring), that is, in particular during leisure time when residents want to spend their time on leisure activities, and motorcyclists want to use their free time for riding a motorbike in nice weather.

Regarding the characteristics of motorcycle sound, high engine revolutions during acceleration, followed by the auditory perception of fast, aggressive riding and a rattle, are reported as the most annoying features of motorcycle sound (Table 8).

Motorcycle noise annoyance, as well as road traffic noise exposure are strongly associated with reported disturbance due to motorcycle noise, in particular with motorcycle noise-related disturbances of outdoor activities as well as with the perceived capacity to cope with motorcycle noise, which supports the conceptualisation of annoyance as including often repeated disturbances by noise and the perceived lack of control and ability to cope with noise [4]. In addition, respondents reporting higher noise sensitivity, less positive and more negative attitudes towards motorcycles, are more annoyed by motorcycle noise. Compared to these correlations, motorcycle noise annoyance is marginally correlated with the (non-source-specific) road traffic noise level *L*_den_, with r < 0.2. (Table 9, see [8] for more details).

#### 3.3.2. Short-Term Exposure–Response Relationships (MotoApp)

Multilevel logistic regressions of hourly vehicle-specific %HA_V_ on source-specific *L*_Aeq,1h_, *L*_AFmax,1h_, and N_60_ were calculated. The parameters of all the GEE logistic regressions are shown in the Supplementary Materials in Tables S1–S3.

**Figure 3:**
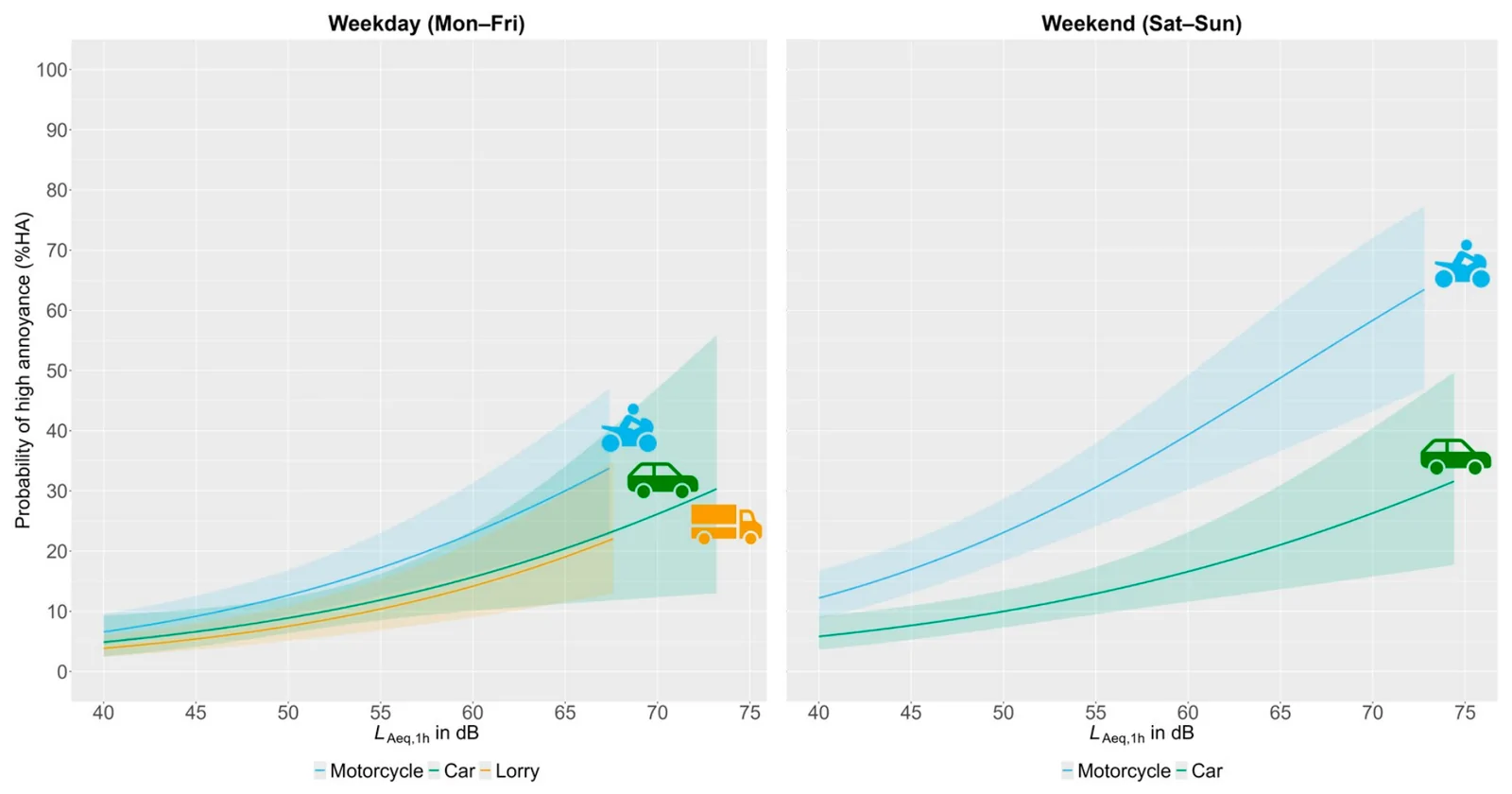
Exposure–response curves for %HA_V_ against *L*_Aeq,1h_ in dB for motorcycles, passenger cars, and lorries based on GEE logistic regressions. %HA curves for lorry noise excluded for weekend due to weekends traffic restrictions for lorries. Parameters of the GEE logistic regressions are shown in the Supplementary Materials in Table S1.

**Figure 4:**
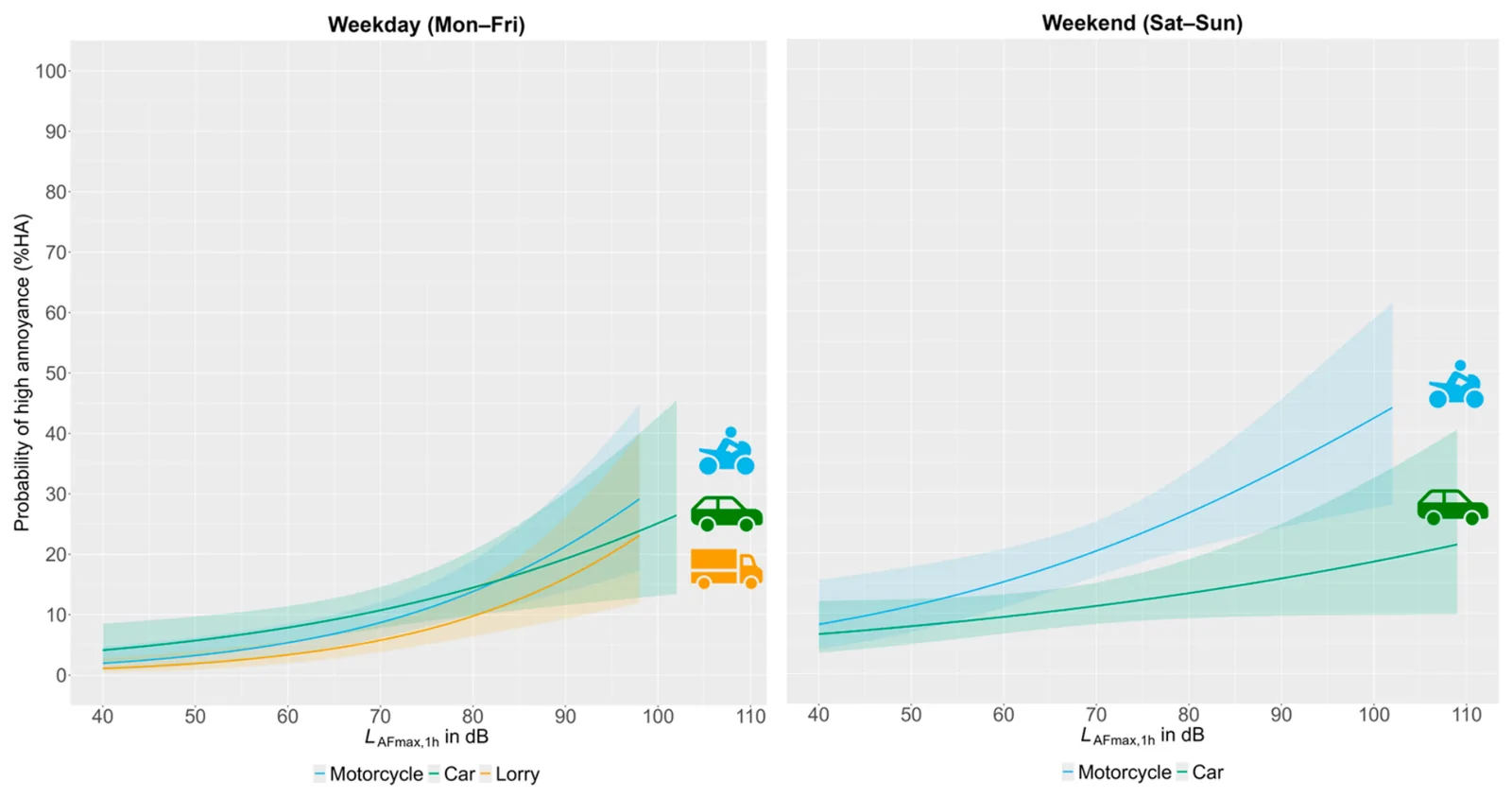
Exposure–response curves for %HA_V_ against source-specific *L*_AFmax,1h_ in dB for motorcycles, passenger cars, and lorries based on GEE logistic regressions. %HA curves for lorry noise excluded for the weekend due to weekend traffic restrictions for lorries. Parameters of the GEE logistic regressions are shown in the Supplementary Materials in Table S2.

The multilevel logistic regression of hourly %HA_V_ on hourly *L*_Aeq,1h_ confirmed that, for a given hourly motorcycle noise level, the probability of a highly annoyed rating was higher on weekends than on weekdays. In contrast, for passenger car noise it is the other way around, i.e., the %HA_V_ curve is slightly higher on weekdays than on weekends (Figure 3), see also Figure 4 in [18]. Furthermore, the higher %HA_V_ curve for motorcycle noise compared to noise from other vehicles is confirmed for hourly annoyance related to hourly source-specific noise exposure (*L*_Aeq,1h_). This is confirmed in an additional exposure–response analysis of %HA_V_ against the hourly maximum sound level, *L*_AFmax,1h_, for weekends. However, for the same hourly *L*_AFmax,1h_ on weekdays, %HA_V_ for motorcycle noise is higher than %HA_V_ for lorry noise, but not different from the %HA_V_ curve against *L*_AFmax,1h_ for noise from passenger cars (Figure 4).

**Figure 5:**
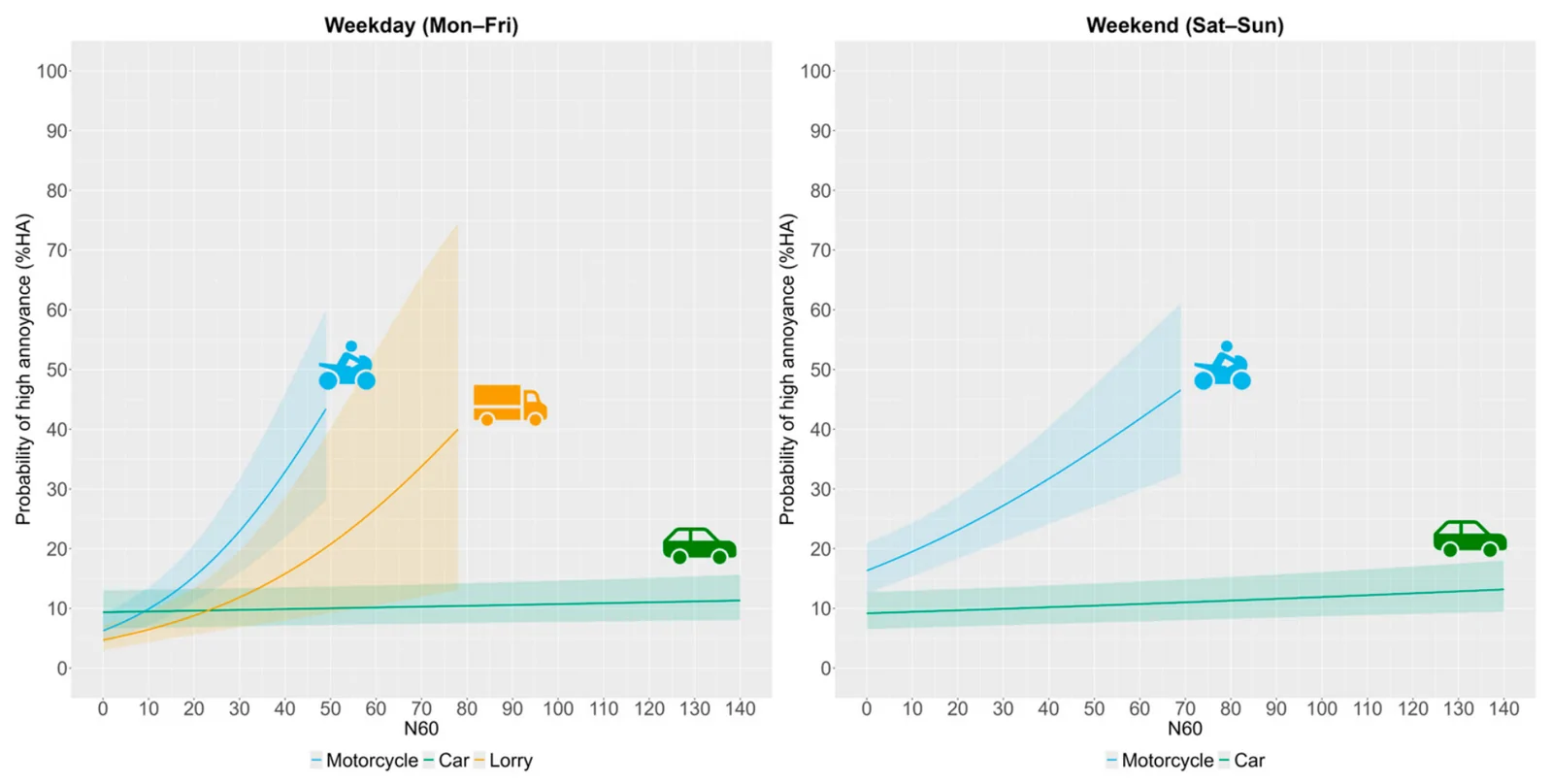
Exposure–response curves for %HA_V_ against N_60_ for motorcycles, passenger cars, and lorries based on GEE logistic regressions. %HA curves for lorry noise excluded for the weekend due to weekend traffic restrictions for lorries. Parameters of the GEE logistic regressions are shown in the Supplementary Materials in Table S3.

Figure 5 depicts the hourly %HA for noise from motorcycles, passenger cars, and lorries on weekdays and (without lorries) on weekends against the number above the threshold of 60 dB *L*_AFmax_ (N_60_). Although with a N_60_ maximum of 771 cars per hour, the N_60_ value of passenger cars reaches about 10 times the maximum N_60_ of motorcycles (Max(N_60_) = 69) and lorries (Max(N_60_) = 78), the N_60_ values in Figure 5 are restricted to a maximum of N_60_ = 140 to allow for better comparisons between the vehicle-specific %HA curves. Again, and more strongly pronounced than in the %HA curves related to *L*_Aeq,1h_ and *L*_AFmax,1h_, for N_60,_ the motorcycle noise-specific %HA curve is higher than the %HA curves for lorry and passenger car noise, both on weekdays and—as compared with passenger cars only—on weekends.

In the Supplementary Materials, Tables S1–S3, the model fit values of the GEE logistic regressions depicted in Figure 3, Figure 4 and Figure 5 indicate different model fits for the predictive models for %HA depending on the noise metrics *L*_Aeq,1h,_, *L*_AFmax_,_1h_ and the N_60_ per hour and the type of day (weekday versus weekend): The AUC values for the *L*_Aeq,1h_ models range between 0.715 and 0.740 for weekdays and between 0.661 and 0.671 for weekends, for the *L*_AFmax,1h_ models between 0.670 and 0.694 for weekdays and between 0.612 and 0.621 for weekends, for the N_60_ models between 0.655 and 0.676 for weekdays, and the AUC value for the N_60_ models for weekends is AUC = 0.610. The AUC values are lower on weekends than on weekdays, which may be because more hourly measurements were taken on Mondays and Fridays than on Saturdays and Sundays. The AUC values, as well as the regression coefficients for the noise exposure variables, are higher for models that use *L*_Aeq,1h_ as an acoustic predictor than for models using *L*_AFmax,1h_ or N_60_. All in all, AUC values between 0.60 and 0.70 indicate low but relevant discrimination of HA vs. non-HA, and AUC values above 0.7 indicate moderate to fair discrimination [33].

Correlation analysis confirms that for more intermittent noise sources, such as lorry and motorcycle noise, compared to the more continuous noise of passenger cars, noise annoyance depends more strongly on the number of ‘loud’ events as operationalised by hourly N_60_ than on the maximum sound level as indicated by repeated-measurements correlation r_rm_ across all weekdays between hourly noise annoyance and noise exposure metrics (Table 10). This correlational structure remains in the subgroups weekdays (Mondays-Fridays) vs. weekend (Saturdays to Sundays) for motorcycle noise, but not consistently for lorry noise. For all vehicles, the correlation coefficients r_rm_ of N_60_ with noise annoyance judgements are on a similar level to the coefficients for *L*_Aeq,1h_ with noise annoyance. For passenger cars, the correlations between noise annoyance and hourly noise exposure metrics are considerably lower than for motorcycles and lorries. Among motorcycle noise metrics, N_60_ correlates most strongly with noise annoyance.

In German-speaking countries, a threshold of 25% of the population being highly annoyed by a given noise level is regarded as a severity level that establishes an unacceptable impact of environmental noise [34]. The threshold values in hourly noise metrics at which the 25–criterion is exceeded are presented separately for motorcycles, passenger cars, and lorries, for weekdays and weekends. At that threshold, motorcycle noise is about 7–8 dB (*L*_Aeq,1h_) more annoying on weekdays than passenger car noise and lorry noise, and 16 dB (*L*_Aeq,1h_) on weekends compared to passenger car noise. Regarding the maximum sound level, the threshold values differ in the same degree between noise from motorcycles and passenger cars on weekdays (*L*_AFmax,1h_: 93 dB for motorcycle, 101 dB for passenger car), but much more on weekends (*L*_AFmax,1h_: 78 dB for motorcycle, >110 dB for passenger car). On weekdays, motorcycle and passenger car noise exposures are about 7 dB more annoying than exposure to lorry noise (*L*_AFmax,1h_). Only a relatively small number of ‘loud’ motorcycles per hour (N_60_ = 32 on weekdays, N_60_ = 25) on weekends leads to the same percentage of highly annoyed people as compared to the number of ‘loud’ passenger cars (N_60_ ≈ 770 on weekdays, N_60_ ≈ 410 on weekends) and the number of lorries (N_60_ = 57 on weekdays); see Table 11. Regarding weekday–weekend differences, Table 11 shows a ‘malus’ of 10 dB in *L*_Aeq,1h_, and 15 dB in *L*_AFmax,1h_ for motorcycle noise. In contrast, no such differences occur for passenger car noise in *L*_Aeq,1h_, and even a bonus of 9 dB in *L*_AFmax,1h_.

## 4. Discussion

### 4.1. The Annoyance Malus of Motorcycle Noise

The central finding of this investigation—that motorcycle noise produces substantially higher rates of high annoyance than road traffic noise overall and noise from other road traffic vehicles at equivalent continuous levels (*L*_den_ in the long-term study, *L*_Aeq,1h_ in the MotoApp study)—is consistent with and extends the existing evidence base. The magnitude of the exposure–response curve (ERC) shift observed in the present study is broadly compatible with that reported by Lechner and colleagues [7] in the Austrian Alps, who documented a shift exceeding 30 dB in *L*_Aeq_ (on summer Sundays at daytime between 6 a.m. and 7 p.m.) relative to other road traffic noise. Taken together, these two independent investigations, conducted in different countries and methodological frameworks, constitute convergent evidence that motorcycle noise carries a systematic annoyance ‘malus’ relative to other road traffic vehicles and mixed traffic at equivalent A-weighted levels.

The acoustic mechanisms underlying this excess annoyance are likely multiple and partially co-occurring. First, the A-weighted equivalent level metric suppresses spectral content outside the principal sensitivity range of the A-weighting network. However, motorcycle noise contains substantial energy at frequencies where the psychoacoustic attributes of sharpness and roughness—known annoyance correlates [10,11]—are prominent. Samani and Altinsoy [10] demonstrated in controlled listening experiments that sharpness and loudness account for variance in annoyance beyond SPL, suggesting that A-weighting-based assessment systematically underestimates the perceptual intensity of motorcycle noise events. Second, the temporal structure of motorcycle noise—characterised by rapid onset, high instantaneous levels, and brief duration—may produce pseudo-startle responses that are not captured by energy-based averaging. The present hourly N-based exposure–response analysis, which shows that event counts above 60 dB *L*_AFmax_ provide additional information beyond *L*_Aeq,1h_ and *L*_AFmax,1h_, is consistent with this hypothesis.

Third, the intermittent nature of motorcycle noise means that periods of relative quiet between successive noise events create repeated attention-orientation cycles, potentially maintaining heightened arousal across the exposure period. The number of ‘loud’ motorcycles (N_60_ events) has a relatively strong impact on noise annoyance, which may reflect the transition from an interrupted quiet environment to a state of continuous intrusion, particularly on weekends. This aligns with qualitative reports by residents of ‘no quiet moment’ during peak weekend periods.

### 4.2. The Weekend Effect: Leisure Expectation and Context-Dependent Sensitivity

The pronounced weekday–weekend differential in the exposure–annoyance relationship—quantified in the present study as a shift of about 10 dB at equivalent hourly *L*_Aeq_ values—is among the most practically significant findings for noise policy purposes. The mechanism is likely grounded in the temporal mismatch between the dominant temporal pattern of motorcycle leisure traffic (concentrated on weekend daytime hours) and the normative expectation of weekend residents regarding acoustic quality during leisure time. Motorcycle noise during these periods coincides with residents’ leisure activities at home, triggering what can be characterised as leisure expectation violation.

This interpretation is consistent with the Alpine findings of Lechner and colleagues [7], who reported similarly concentrated weekend and summer weekend patterns, and noted that motorcycle annoyance was largely independent of background car traffic noise—implying that residents evaluate motorcycle noise relative to their expectations for a quiet leisure environment rather than relative to the ambient noise floor. Our data allows testing the assumption of Lechner and colleagues [7] regarding the independence of motorcycle noise annoyance from background car (and lorry) traffic noise. In sensitivity analyses we calculated three multiple GEE models for source-specific %HA due to motorcycle, passenger car, and lorry noise, respectively, with the predictors *L*_Aeq,1h,motorcycle_, *L*_Aeq,1h,car_, *L*_Aeq,1h,lorry_ and the annoyance scores for noise from the respective other vehicle classes separately for the total sample and separately for weekdays (Mon–Fri) and weekends (with %HA due to lorry noise excluded in the weekend analysis; see Supplementary Materials, Tables S4–S6). For comparison, Tables S4–S6 include the respective GEE models with the single source-specific *L*_Aeq,1h_ as predictor. In sum, the Tables S4–S6 show the following:

- The source-specific %HA for motorcycle, passenger car, and lorry noise were either not statistically associated or negatively associated with the *L*_Aeq,1h_ of the other road traffic noise sources. This suggests that exposure to noise from other vehicle types does not add to annoyance from motorcycle noise, and vice versa. The partly statistically significant negative regression coefficients for the other vehicles’ exposure (*L*_Aeq,1h_) likely reflect multicollinearity among exposure variables (VIF values up to 6.6).
- Consistent with this, adding source-specific exposure variables for other vehicle types did not attenuate the regression coefficient of the source-specific *L*_Aeq,1h_ for the respective source-specific %HA. Overall, there is little evidence that exposure to one source increases annoyance attributed to another source. This pattern was observed in the overall analysis (S4) as well as in the analyses grouped by weekdays (S5) and weekends (S6).

Across all analyses (overall, weekdays, weekends), %HA for motorcycle noise was positively associated with annoyance due to passenger car noise, and vice versa, with a similar reciprocal pattern for passenger car and lorry noise. These cross-source associations are more consistent with a halo effect than with spillover, suggesting that a shared overall evaluation of road traffic noise may influence annoyance ratings for individual sources rather than annoyance from passenger car, motorcycle, or lorry noise directly reinforcing one another. The assumption of Lechner and colleagues that the motorcycle noise is evaluated relative to residents’ expectations for a quiet home leisure environment on weekends is also supported by conclusions of other authors that residents expect a quiet and restful environment at home allowing for recreation especially after working days [35,36,37]. Other authors attribute the higher annoyance on weekends to more people spending more time at home on weekends than on weekdays, i.e., they are longer exposed to environmental noise at home on weekends and bank holidays than on other days of the week [38]. Both explanations are plausible and may complement each other.

Motorcycle noise and its perception are strongly influenced by riders’ driving behaviour. Findings from the long-term study on the annoying features of motorcycle sound suggest that individuals perceive not only the acoustic signal itself, but also the behaviour that generates it. Consequently, certain riding styles—particularly those perceived as reckless—constitute an “audible behaviour” that shapes noise perception, and, thus, annoyance. In this sense, environmental noise should be understood not only as a physical phenomenon but also as a social one. Within this framework, Stallen regarded noise exposure as an interpersonal act, summarised by the concept of “you expose me.” [39].

From a methodological perspective, the present findings highlight the inadequacy of annual rating levels, such as the *L*_Aeq_ for daytime or the *L*_den_ as an exposure indicator for motorcycle-noise assessment. An annual equivalent continuous noise level by design aggregates acoustic energy across all days of the year, including periods when motorcycles do not contribute to the noise climate (winter, weekday nights, morning periods). A metric based on annual energy averaging over a predominantly car-traffic baseline will systematically underestimate the peak exposure experienced by residents on typical summer Sundays. The short-term MotoApp methodology adopted in this study captures these temporal dynamics at the hourly level. It demonstrates that the exposure–annoyance relationship operates on a time scale of hours, days, and seasons, not years.

### 4.3. Non-Acoustic Factors: Attitudes, Sensitivity, and the Role of Attributional Appraisal

The high correlations of source-specific activity disturbances with motorcycle noise annoyance and the fact that these variables also correlate with the noise level (*L*_den_) confirm the view that these constructs are elements of the concept of annoyance [4]. Authors also regarded the capacity to cope with noise as a further element of noise annoyance [4,40]. In this study, the capacity to cope with motorcycle noise highly correlated (r = −0.73) with motorcycle noise annoyance but, contrary to the disturbance variables, not statistically significant with the road traffic noise exposure (r = −0.09), indicating that the perceived capacity to cope with motorcycle noise is not an element of the annoyance concept, but forms a non-acoustical factor that contributes to the prediction of long-term noise annoyance. The further dominance of a negative attitude towards motorcycle riders as a non-acoustic predictor of long-term annoyance—exceeding the contribution of noise sensitivity in the final regression model—is of both theoretical and practical importance. This finding, which is supported by similar results of the Austrian Alpine Motorcycle study [7], extends to the motorcycle noise domain a well-established principle from research on other sources of environmental noise: that the appraisal of the noise source, rather than acoustic exposure alone, is the most important determinant of noise annoyance [14,39]. In the motorcycle noise context, negative attitudes reflect attributional cognitions, specifically, the perception that motorcycle riders are reckless, dangerous, and inconsiderate, which leads residents to interpret the same acoustic event as more aversive than they would if the source were perceived as legitimate or socially valued. The finding that a positive attitude towards motorcycling partially mitigated annoyance at equivalent exposure levels reinforces this interpretation. Residents who associated motorcycling with positive values (freedom, enjoyment of landscape) reported lower annoyance, suggesting that attitudinal salience operates bidirectionally. These data have potentially important implications for noise management: purely technical interventions that reduce noise levels without addressing the perceived legitimacy of motorcycle use may yield smaller annoyance reductions, because the attitudinal component of annoyance will persist.

Noise sensitivity, the third-strongest non-acoustic predictor, operated as expected from the general noise annoyance literature: more sensitive individuals reported higher annoyance at equivalent exposure levels [14,41]. The self-report sensitivity item used in the present study, consistent with other recent socio-acoustic investigations, is a validated predictor of annoyance across source types. However, it cannot distinguish between constitutional sensitivity and context-specific acquired sensitisation to motorcycle noise.

### 4.4. Implications for Noise Policy and Regulatory Standards

The present findings have several direct implications for noise policy in Germany and at the European level. First, the data demonstrate that source-specific exposure assessment is necessary for motorcycle-dominated leisure routes: standard road traffic noise mapping based on *L*_den_ systematically underestimates the community burden of annoyance from motorcycle noise in such settings. This suggests that local noise action plans prepared under the Environmental Noise Directive (2002/49/EC) [3] would benefit from more source-specific noise mapping, particularly, where, for example, motorcycle leisure traffic is a significant contributor. This could include using hourly equivalent levels and event-count metrics to characterise the noise burden imposed by motorcycle leisure traffic adequately. Annual *L*_den_-based levels on mixed-traffic streams are insufficient for this purpose. For example, as is the case in Germany for aircraft noise according to the Aircraft Noise Act, from a noise health effect perspective, rating levels for motorcycle noise should be calculated for the six busiest months (equivalent to the warmer season) in order to minimise the underestimation of motorcycle noise.

Second, the 25% HA criterion—widely used as an environmental quality target in noise policy in German-speaking countries—maps to a motorcycle-specific hourly *L*_Aeq_ of approximately 52 dB on weekends and 62 dB on weekdays in the present dataset. These values are substantially below *L*_den_ values at which standard road traffic curves reach 25% HA [4]. Lechner and colleagues [7] similarly derived lower threshold values for Alpine motorcycle routes using source-specific ERCs. The convergence of two independently conducted European studies on a substantially lower threshold for motorcycle noise lends weight to the argument for source-specific limit values along popular motorcycle routes.

As in this study as well as in the Austrian motorcycle noise study [7], motorcycle noise annoyance was higher on weekends, particularly Sundays, stricter exposure standards or critical limit values for motorcycle noise could be imposed for weekends.

Third, the N_60_-based analysis suggests that event-count metrics—supplementing energy-based *L*_Aeq_—provide additional information about noise annoyance risk that is not captured by equivalent continuous levels alone. A limit of 25 motorcycle events per hour exceeding 60 dB(A) on weekends (32 on weekdays) may serve as a complementary operational criterion for assessment and management purposes. To minimise the number of loud motorcycle noise events, noise regulation could include implementing a speed limit [42] or even a temporary ban on motorcycling on routes with heavy motorcycle traffic [43]. These interventions could be implemented especially for weekends or Sundays in warmer seasons, only, given the higher noise annoyance on those days. A motorcycling ban could be imposed based on the volume and noise level of motorcycle traffic, as assessed through traffic and noise monitoring along the routes. Similar considerations apply in Austria as a result of Lechner and colleagues’ study of motorcycle noise [7]. Such a ban was implemented for loud motorcycles (defined by near-field sound levels above 95 dB) in summer 2020 in the Reutte region in Tyrol, Austria [43] and evaluated [44]. The main results were that, due to the motorcycling ban and the COVID-19 pandemic, motorcycle traffic on summer Sundays decreased by an average of 36% in 2020 compared to 2017, equivalent to a 2 dB *L*_Aeq_ reduction. In line with this, an evaluation survey of 250 residents from 19 communities in the Reutte region found that the percentage of highly annoyed people due to motorcycle noise halved to 29%, compared with the 2019 motorcycle noise study [7,44]. Most respondents (58%) supported riding bans on motorbikes with near-field sound levels exceeding 95 dB(A). 

Fourth, the prominent role of attitudinal factors suggests that communication-based and participatory governance interventions—involving motorcycle rider communities and residents in route management discussions, creating designated leisure routes with agreed noise-behaviour codes, or incentivising the uptake of quieter motorcycles—may yield community benefit beyond what can be achieved through regulatory emission limits alone. Dendale and Münzel [20] have recently called for stronger regulatory attention to motorcycles within European environmental health policy. However, in addition to stronger regulatory measures addressing motorcycle noise, effective noise management strategies should address both the acoustic and attitudinal dimensions of residents’ noise annoyance, combining technical emission-reduction measures with the above-mentioned engagement and governance approaches.

### 4.5. Comparison with International Literature

The present study shares key methodological features with the Alpine study of Lechner et al. [7]: both used address-based community samples alongside popular motorcycle leisure routes, source-specific exposure assessment, and ISO/TS 15666-compliant annoyance measurement. The broadly comparable ERC shift magnitudes across these two independent studies, despite differences in topography, study design (e.g., long-term only vs. mixed long-term and short-term annoyance assessment), strengthen the inference that motorcycle noise in scenic leisure contexts exerts a systematically higher annoyance per unit *L*_Aeq_ than standard models predict. At the same time, differences in the magnitude of attitude effects and in the specific threshold values derived across settings suggest that local context modulates the precise shape of the ERC.

In a broader international context, the findings also resonate with traffic noise research from motorcycle-intensive urban environments in South-East Asia [19], where high proportions of motorcycle traffic have been associated with exceedances of residential noise guideline values. However, the mechanism of excess annoyance in leisure-route contexts is mediated by temporal expectations, attitudinal appraisal, and the salience of individual high-level events. Therefore, it appears qualitatively distinct from the annoyance attributable to the sheer density of motorcycle traffic in high-volume urban settings, underscoring the importance of source-context specificity in noise assessment.

### 4.6. Strengths and Limitations

This investigation offers several methodological strengths. The combined use of long-term retrospective survey data and prospective ESM assessments within the same population constitutes a novel methodological approach in motorcycle noise research, enabling the characterisation of both chronic and acute annoyance dynamics. The address-level acoustic modelling, in the ESM study based on measurements, provides substantially higher exposure resolution than studies relying on total road traffic *L*_den_ alone. The large, geographically diverse sample across five study areas within a single region strengthens internal and external validity in the study context. The standardised use of ISO/TS 15666 annoyance scales ensures international comparability.

Key limitations must be acknowledged. The cross-sectional design precludes causal inference about the direction of the attitude–annoyance relationship; reverse causation (high annoyance leading to negative attitudes) and confounding by shared third variables cannot be ruled out. The study was conducted only in summer 2022, precluding year-round exposure characterisation; given the pronounced seasonality of motorcycle traffic, winter and spring exposures are not represented. The achieved response rate of 19% in the long-term study raises the possibility of self-selection bias, particularly if highly annoyed residents were more likely to participate, which would upwardly bias %HA estimates. Unfortunately, small-scale census data for the study areas were not available for analysis on potential demographic distortions.

In the study on long-term noise responses, no vehicle-specific sound metrics were available; thus, the *L*_den_ for total road traffic noise was related to vehicle-specific %HA_V_ in the exposure–response analysis. This limits comparison of vehicle-specific %HAV, as the *L*_den_ for road traffic noise includes noise exposure from all vehicle classes. For the interpretation of the higher %HA_V_ for motorcycle noise, the information may be helpful that in most study areas the average daily motorcycle traffic reached about 10% (on weekends: up to 15%) of the total traffic volume (in Oppenau: 27% on all weekdays and 38% on weekends). That is, the number of motorcycles contributes only a minor amount to the average daily traffic volume, which, together with the exposure–response curves for %HA, suggests a relatively greater relevance of this vehicle class to road traffic noise annoyance compared to other vehicle classes. This is confirmed by the short-term results of the MotoApp substudy, which examined the relationship between vehicle-specific noise exposure metrics and annoyance.

The AUC values of the GEE models for the short-term %HA ranged between 0.6 and 0.8, indicating a low to fair discrimination of participants being highly vs. not highly annoyed, depending on the noise exposure metric chosen as a single predictor and the type of day (weekday versus weekend) [33]. Still, the exposure–response curves can be interpreted as regression coefficients, and their *p*-values demonstrate a consistent, statistically significant association between noise exposure and %HA across conditions. We distinguish between predictive and explanatory approaches to conducting exposure–response modelling [45,46]. With our exposure–response models, we aim to follow the explanatory (“risk factor”) approach, i.e., to estimate the effect of the risk factor “noise exposure” on noise annoyance, while acknowledging that a single acoustic predictor has limited discriminative ability for %HA given the considerable impact of non-acoustic factors on annoyance [4,14,15]. The different metrics have distinct AUC values and regression coefficients, indicating that *L*_Aeq,1h_ seems to be a noise exposure indicator slightly more strongly associated with %HA. This is plausible, as the *L*_Aeq_ is based on both the intensity of noise events (level) and the number of noise events.

The MotoApp subsample was younger on average than non-participants, though sensitivity analyses did not support materially different profiles of annoyance or sensitivity. Annual *L*_Aeq_ values for the long-term survey were modelled over 12 months rather than measured directly, as in the short-term study, introducing methodological differences and resulting uncertainty in the assessments between the long-term and short-term studies.

It cannot be ruled out that the sample participating in the long-term study and part of them in the MotoApp study are different in personal characteristics from the underlying population in the study area, and that part of these differences affects the noise annoyance judgements. Whether the sample in this study is more annoyed by motorcycle noise than the population in the study areas, or in Baden-Wuerttemberg and Germany, respectively, cannot be answered as neither regional nor nationwide information about motorcycle noise annoyance is available.

Regarding noise sensitivity, 4% of the study participants (long-term study) reported being not at all sensitive, 12% little, 22% moderately, 31% rather, and 31% very sensitive to noise. In their Austrian motorcycle noise study with 571 respondents, Lechner and colleagues [47] used a similar 5-point rating scale to assess noise sensitivity via a one-time self-report. In their study, 12% are not at all sensitive to noise, 18% slightly, 46% moderately, 17% very, and 7% extremely sensitive to noise. In a Dutch study on noise sensitivity with a sample of 5806 subjects, 12.5% reported being highly noise-sensitive (the highest category on a 5-point scale) [48]; in this study, the proportion is 31%. In the German NORAH survey with a sample of 26,607 participants from four airport regions [49], 20% reported being highly noise-sensitive (the highest score on a single-item 4-point rating scale). Compared with these two studies, the sample in this study appears to have a higher proportion of highly sensitive individuals. Whether this is a selection bias or reflects the noise sensitivity in the underlying population of the study areas cannot be decided.

In this sample, 80% reported being at least slightly annoyed by road traffic noise; in regular biannual surveys of the German population by the German Environment Agency, it has been 67% to 77% since 2016. However, the sites in this study are not representative of Germany, as they are specifically selected rural areas in a landscape that attracts many tourists to the region by car, bus, train, and motorbike. Thus, the annoyance reported by the sample in this study may differ from judgements of people living in urban areas.

In an additional sensitivity analysis, the robustness of the exposure–response curves for %HA from motorcycle, passenger car, and lorry noise, respectively, was tested against different distributions of known and unknown individual characteristics in the sample. For this, the sample of participants taking part in both study parts, the long-term and the MotoApp study, was used. The GEE models for %HA against *L*_Aeq,1h_ for motorcycle, passenger car, and lorry noise were re-calculated using cluster bootstrap resampling with *N*_bootstrap_ = 5000 replications with replacement. The pooled sample of measurements (weekday and weekend) was used to assess the robustness of the exposure–response relationship. Bootstrapping does not directly address non-response bias; however, it reflects the internal stability of participant composition [50,51]. For example, a potential annoyance effect of people with low socio-economic status (SES) being under-represented in the study cannot be tested with bootstrapping. Nevertheless, the robustness check by means of bootstrapping indicates whether changes in the frequency of low-SES participants in the bootstrap samples affect the stability of the exposure–response curves and indirectly informs about the extent of the effect of a potential bias due to individual characteristics underlying the decision on study participation. The results presented in Table S7, particularly the bias score values close to zero, indicate a high internal stability of the exposure–response relationships with respect to participant composition.

## 5. Conclusions

This mixed-methods socio-acoustic field study demonstrates that motorcycle noise along popular leisure routes in southwest Germany produces substantially higher rates of residential noise annoyance than generic road traffic exposure–response models would predict. Approximately half of the surveyed resident population was highly annoyed by motorcycle noise—a prevalence exceeding that predicted by standard reference curves at comparable equivalent continuous levels. Source-specific exposure–response functions for motorcycles are markedly shifted to lower levels than those of other road traffic sources, with the weekend shift relative to cars reaching 16 dB in *L*_Aeq,1h_ at the 25% HA_V_ threshold.

Non-acoustic individual factors—particularly the perceived capacity to cope with motorcycle noise, negative attitudinal appraisal of motorcycle riders, and noise sensitivity—are significant predictors of annoyance over and above the noise exposure. The experience-sampling component of the study reveals that short-term (hourly) noise annoyance is highest on weekends.

The findings of this study particularly refer to the situation in rural areas along attractive motorcycle routes. It is plausible that judgements about motorcycle noise may differ in urban areas. Future research on road traffic noise is needed that also includes participants living in urban areas and assesses exposure and responses to road traffic noise vehicle class-specifically, as was done in this study. However, at least in Germany and Austria, there are several rural regions with a landscape attracting motorcyclists, such as the Eifel (west/southwest of Germany), Sauerland (west-central Germany), and Außerfern (northwestern Tyrol, Austria), where an increasing number of communities report suffering from unbearable motorcycle noise, particularly in warmer seasons. It is expected that the results of this study can be generalised to such regions. However, future research should verify whether the present findings generalise to other European leisure-route contexts. Furthermore, future studies should investigate the health implications of chronic high annoyance in motorcycle-noise-affected communities and explore the feasibility and acceptance of alternative exposure metrics—including psychoacoustic loudness and sharpness—as supplements to A-weighted level in regulatory practice. Longitudinal panel designs would enable causal inference regarding the attitude–annoyance relationship and the long-term trajectory of noise annoyance in response to traffic management interventions.

## Figures and Tables

**Table 1:** Averaged hourly road traffic volume in the study areas based on traffic census data of the Ministry of Transport Baden-Wuerttemberg from 2020 and 2021.

	Average Daily Pass-Bys Across All Days per Week					Average Daily Pass-Bys During Weekends				
	Total	Passenger Car	Lorry	Motorcycle		Total	Passenger Car	Lorry	Motorcycle	
	ADTV *	ADTV *	ADTV *	ADTV *	%	ADTV *	ADTV *	ADTV *	ADTV *	%
Engen	9569.71	8743.43	334.43	491.86	5.1%	8335.50	7415.00	91.00	829.50	10.0%
Oppenau	1245.23	863.54	44.77	336.92	27.1%	1788.00	1077.00	24.00	687.00	38.4%
Gernsbach	3942.22	3507.56	86.44	348.22	8.8%	2128.50	1809.50	15.00	304.00	14.3%
Gaggenau	4283.86	4008.29	59.14	216.43	5.1%	3957.50	3536.50	18.50	402.50	10.2%
Güglingen	10,491.86	9942.14	272.29	277.43	2.6%	7836.50	7419.00	58.00	359.50	4.6%

* ADTV: Average daily traffic volume (pass-bys per 24 h).

**Table 2:** Study design of the MotoApp study.

Group	Days	5-Day Period	Notifications (Assessment Times)					
A	Wednesday– Sunday	1	9 a.m.	11 a.m.	1 p.m.	3 p.m.	5 p.m.	7 p.m.
	Friday– Tuesday	2	10 a.m.	12 p.m.	2 p.m.	4 p.m.	6 p.m.	8 p.m.
B	Wednesday– Sunday	1	10 a.m.	12 p.m.	2 p.m.	4 p.m.	6 p.m.	8 p.m.
	Friday– Tuesday	2	9 a.m.	11 a.m.	1 p.m.	3 p.m.	5 p.m.	7 p.m.

**Table 3:** Descriptives of the samples of the long-term survey and MotoApp survey.

Variable	Category/ Description	Long-Term Survey (*N* = 493)			MotoApp Subsample (*N* = 213)		
		*n*	%		*n*	%	
Gender	male	240	48.7%		101	47.4%	
	female	240	48.7%		110	51.6%	
	diverse	0	0.0%		0	0.0%	
	no resp.	13	2.6%		2	0.9%	
Age (in years)	18–39	122	24.7%		79	37.1%	
	40–59	175	35.5%		88	41.3%	
	60–79	160	32.5%		39	18.3%	
	80+	23	4.7%		3	1.4%	
	no resp.	13	2.6%		4	1.9%	
House ownership	owners	374	75.9%		147	69.0%	
	tenants	118	23.9%		66	31.0%	
	no resp.	1	0.2%		0	0.0%	
Motorcycle driver’s licence	yes	158	32.0%		69	32.4%	
	no	331	67.1%		144	67.6%	
	no resp.	4	0.8%		0	0.0%	
		*M*	*SD*	*n valid*	*M*	*SD*	*n valid*
Age	(in years)	52.7	17.2	480	46.3	16.3	209
Length of residence	(in years)	19.6	16.7	479	16.3	15.1	210
Socio-economic status	(1 = low, 7 = high)	4.2	1.0	477	4.2	0.9	212
Noise sensitivity	(1 = low, 5 = high)	3.7	1.1	490	3.7	1.1	213
Satisfaction with neighbourhood	(1 = low, 5 = high)	4.2	0.9	475	4.1	0.9	212
Satisfaction with dwelling	(1 = low, 5 = high)	4.4	0.8	483	4.3	0.9	212

*N* = sample size; *n* = number of responses; % = percentage; *M* = mean; *SD* = standard deviation; no resp. = no response, missing.

**Table 4:** Descriptives of annoyance and exposure variables of the long-term and MotoApp studies for motorcycle, passenger car, and lorry noise.

Variable	Sample/ Vehicle	*n* *	*M*	*SD*	*Min*	*Max*
Long-term study						
Annoyance total sample	Motorcycle	488	3.1	1.5	1	5
	Passenger car	479	2.5	1.1	1	5
	Lorry	475	2.3	1.2	1	5
Annoyance MotoApp participants	Motorcycle	212	3.0	1.5	1	5
	Passenger car	212	2.4	1.1	1	5
	Lorry	212	2.3	1.2	1	5
*L*_den_	Long-term	493	53.8	7.8	36.9	74.2
	MotoApp	213	54.9	7.6	36.9	71.9
MotoApp study, total						
Annoyance	Motorcycle	4040	1.9	1.2	1	5
	Passenger car	4036	2.0	1.1	1	5
	Lorry	4037	1.4	0.7	1	5
*L*_Aeq,1h_	Motorcycle	3610	43.8	9.5	20.1	72.9
	Passenger car	3622	49.9	7.8	27.2	74.4
	Lorry	3115	39.8	10.0	20.0	68.4
*L*_AFmax,1h_	Motorcycle	3098	68.4	10.8	50	102
	Passenger car	3085	67.1	10.6	50	109
	Lorry	2839	66.7	10.7	50	106
N_60_ /hour	Motorcycle	3098	9.1	12.0	0	69
	Passenger car	3085	71.2	109.8	0	771
	Lorry	2839	4.8	8.2	0	78
MotoApp study, weekdays (Monday–Friday)						
Annoyance	Motorcycle	2160	1.7	1.1	1	5
	Passenger car	2157	1.9	1.0	1	5
	Lorry	2159	1.5	0.8	1	5
*L*_Aeq,1h_	Motorcycle	1918	42.0	9.4	20.2	67.5
	Passenger car	1930	50.1	7.8	27.9	73.2
	Lorry	1867	42.4	9.3	20.2	67.8
*L*_AFmax,1h_	Motorcycle	1602	67.7	10.6	50	98
	Passenger car	1639	66.8	10.4	50	102
	Lorry	1653	67.9	10.5	50	98
N_60_ /hour	Motorcycle	1602	6.5	8.2	0	49
	Passenger car	1639	76.7	118.6	0	771
	Lorry	1653	7.0	10.1	0	78
MotoApp study, weekend (Saturday–Sunday)						
Annoyance	Motorcycle	1880	2.1	1.3	1	5
	Passenger car	1879	2.0	1.1	1	5
	Lorry	1878	1.2	0.5	1	5
*L*_Aeq,1h_	Motorcycle	1692	45.9	9.2	20.1	72.9
	Passenger car	1692	49.8	7.9	27.2	74.4
	Lorry	1248	35.9	9.8	20.0	68.4
*L*_AFmax,1h_	Motorcycle	1496	69.2	10.9	50	102
	Passenger car	1446	67.4	10.8	50	109
	Lorry	1186	65.0	10.6	50	106
N_60_ /hour	Motorcycle	1496	12.0	14.6	0	69
	Passenger car	1446	64.9	98.6	0	635
	Lorry	1186	1.7	2.2	0	15

* Number of observations (*n*) in the long-term study refer to the number of participants, in the MotoApp study to the number of valid hourly measurements during the field period; *M* = mean; *SD* = standard deviation; *Min* = minimum; *Max* = maximum.

**Table 5:** Prevalence of annoyance due to several sources of road traffic noise [8].

Source	*n*	Thinking About the Last 12 Months, When You Are Here at Home, How Much Does Noise from the Following Noise Source Bother, Disturb, or Annoy You?					*HA_V_*	*M*	*SD*
		(1) Not at All	(2) Slightly	(3) Moderately	(4) Very	(5) Extremely			
Road traffic overall	486	18.7%	28.6%	27.6%	20.4%	4.7%	25.1%	2.6	1.1
Car	479	23.4%	29.2%	29.0%	15.9%	2.5%	18.4%	2.5	1.1
Lorry	475	32.2%	26.7%	21.7%	14.9%	4.4%	19.3%	2.3	1.2
Coach	467	56.1%	21.8%	15.8%	4.9%	1.3%	6.2%	1.7	1.0
Agricultural traffic	482	47.5%	24.3%	19.5%	5.8%	2.9%	8.7%	1.9	1.1
Motorcycle	488	21.7%	15.6%	16.2%	24.8%	21.7%	46.5%	3.1	1.5

*n* = number of participants; *HA_V_* = highly annoyed; *M* = mean; *SD* = standard deviation.

**Table 6:** Coefficients of logistic regressions for the probability of high annoyance (HA_V_) due to noise from several vehicles.

Variable	Parameter	Road (Overall)	Motorcycle	Passenger Car	Lorry	Coach	Agricultural Traffic
Intercept	*B*	−6.656	−2.364	−5.784	−8.947	−7.635	−5.971
	*p*	<0.001	<0.001	<0.001	<0.001	<0.001	<0.001
*L*_den_ road	*B*	0.101	0.041	0.078	0.134	0.087	0.065
	*p*	<0.001	<0.001	<0.001	<0.001	<0.001	0.001
*N*		485	487	478	474	466	481
*AIC*		497	664.8	433.9	396.3	207.8	278.5
*BIC*		505.4	673.1	442.2	404.6	216.1	286.8
*Log. Likelihood*		−246.519	−330.379	−214.950	−196.159	−101.908	−137.231
*RMSE*		0.41	0.49	0.38	0.36	0.24	0.28

*B* = coefficient; *p* = significance level; *AIC* = Akaike Information Criterion; *BIC* = Bayesian Information Criterion; *RMSE* = Root Mean Squared Error.

**Table 7:** Most annoying time periods per day, week, and season (multiple responses of 493 participants) [8].

Time of Day			Day of Week			Season		
	***n***	**%**		***n***	**%**		***n***	**%**
Morning	60	11.4%	Monday– Friday	167	33.9%	Spring	149	28.8%
Noon	66	12.5%	Saturday	94	19.1%	Summer	320	61.9%
Afternoon	217	41.1%	Sunday, holiday	232	47.1%	Autumn	41	7.9%
Evening	124	23.5%				Winter	7	1.4%
Night	61	11.6%						
Total	528	100.0%	Total	493	100.0%	Total	517	100.0%

*n* = number of responses; % = percentage.

**Table 8:** Annoying characteristics and features of motorcycle sound.

Features of Motorcycle Sound	*n*	*M*	*SD*
high revolutions while accelerating	483	3.5	1.4
fast and aggressive riding	480	3.0	1.4
rattle	477	3.0	1.3
groups of motorcycles	479	2.8	1.4
low frequencies “humming”	477	2.6	1.3

*n* = number of participants; *M* = mean; *SD* = standard deviation.

**Table 9:** Correlation r for motorcycle noise annoyance and road traffic noise level *L*_den_ road with several variables of disturbances, attitudes and personal disposition (see also [8]).

Variable	Motorcycle Noise Annoyance	*L*_den_ Road
*L*_den_	0.190	1.000
Disturbances due to motorcycle noise …		
when having a conversations or phone call	0.690	0.184
while listening to radio/music or watching television	0.669	0.197
while reading, thinking or concentrating	0.706	0.154
while relaxing and taking a rest after work	0.749	0.137
when socialising at home or when having visitors	0.686	0.156
when staying and relaxing outdoors	0.832	0.135
when having conversations outdoors	0.807	0.178
Capacity to cope with motorcycle noise	−0.730	−0.086 #
Noise sensitivity	0.502	−0.028 #
Pos. attitudes toward motorcycles	−0.571	−0.062 #
Neg. attitudes toward motorcycles	0.651	0.002 #

# Correlation coefficient statistically not significant (*p* > 0.05). For all other coefficients: *p* < 0.05.

**Table 10:** Repeated-measurements correlation r_rm_ between source-specific noise exposure metrics and noise annoyance.

Hourly Noise Exposure Metric	Hourly Annoyance Due to Noise From		
	Motorcycle	Passenger Car	Lorry
Total			
*L*_Aeq,1h_	0.220	0.067	0.222
*L*_AFmax,1h_	0.121	0.023 ^ns^	0.167
N_60_	0.241	0.059	0.240
Weekdays (Monday–Friday)			
*L*_Aeq,1h_	0.105	0.094	0.154
*L*_AFmax,1h_	0.083	0.034 ^ns^	0.100
N_60_	0.151	0.056 *	0.073
Weekend (Saturday–Sunday)			
*L*_Aeq,1h_	0.194	0.033 ^ns^	0.051 ^ns^
*L*_AFmax,1h_	0.073	0.007 ^ns^	0.053 ^ns^
N_60_	0.215	0.065 *	0.076 *

Unless marked otherwise, correlation coefficients are statistically significant at *p* < 0.01. * *p* < 0.05; ns not significant (*p* ≥ 0.05).

**Table 11:** Threshold values in hourly noise metrics of motorcycle, passenger car, and lorry noise at which 25% are highly annoyed (%HA_V_).

Noise Metrics	Motorcycle	Passenger Car	Lorry
	Twenty-Five Percent Are Highly Annoyed (HA_V_) at the Level of …		
Weekdays (Monday–Friday)			
*L*_Aeq,1h_	62 dB	69 dB	70 dB
*L*_AFmax,1h_	93 dB	101 dB	~100 dB
N_60_	32	770	57
Weekend (Saturday–Sunday)			
*L*_Aeq,1h_	52 dB	68 dB	--
*L*_AFmax,1h_	78 dB	>110 dB	--
N_60_	25	410	--

## Data Availability

Data available on request from the authors due to restrictions (privacy, ensuring compliance with data protection guidelines).

## Supplementary Materials

The following supporting information can be downloaded at: https://www.mdpi.com/article/10.3390/ijerph23080966/s1, Table S1: Results of GEE logistic regressions for %HA on *L*_Aeq,1h_; Table S2: Results of GEE logistic regressions for %HA on *L*_AFmax,1h_; Table S3: Results of GEE logistic regressions for %HA on N_60_; Table S4: Sensitivity analyses: Results of GEE logistic regressions for source-specific %HA with *L*_Aeq,1h,motorcycle_, *L*_Aeq,1h,car_ *L*_Aeq,1h,lorry_ and the annoyance scores for noise of the respective other vehicle classes; Table S5: Sensitivity analyses: Results of GEE logistic regressions for source-specific %HA with *L*_Aeq,1h,motorcycle_, *L*_Aeq,1h,car_, *L*_Aeq,1h,lorry_ and the annoyance scores for noise of the respective other vehicle classes (weekdays); Table S6: Sensitivity analyses: Results of GEE logistic regressions for source-specific %HA with *L*_Aeq,1h,motorcycle_, *L*_Aeq,1h,car_ *L*_Aeq,1h,lorry_ and the annoyance scores for noise of the respective other vehicle classes (weekend); Table S7: Cluster bootstrap of the source-specific exposure–response coefficients (hourly *L*_Aeq,1h_), MotoApp study, total period (weekdays and weekends).

## Abbreviations

The following abbreviations are used in this manuscript:

%HA	Percentage highly annoyed
%HA_V_	Percentage highly annoyed based on the verbal 5-point annoyance scale as defined in ISO/TS 15666:2021, whereas those judging their annoyance as very (4) and extremely (5) are regarded as being highly annoyed
ANCOVA	Analysis of covariance
DALY	Disability-adjusted life years
dB	Decibel
EEA	European Environment Agency
END	European Environmental Noise Directive 2002/49/EC
ERC	Exposure–response curve
ESM	Experience-sampling method
GDPR	General Data Protection Regulation (EU) 2016/679
GEE	Generalised estimation equation
ICBEN	International Commission on Biological Effects of Noise
*L*_Aeq,1h_	Vehicle-specific A-weighted equivalent continuous sound pressure level for a period of one hour
*L*_AFmax,1h_	Vehicle-specific maximum A-weighted sound pressure level of all events per hour
*L*_den_ road	Day–Evening–Night rating level for a 24 h day for road traffic noise with ‘day’ referring to the period from 6 a.m. to 6 p.m., ‘evening’ to the period from 6 p.m. to 10 p.m. and ‘night’ to the period from 10 p.m. to 6 a.m. In *L*_den_, a 5 dB penalty is added to the rating level for the evening (*L*_evening_), and 10 dB to the rating level for the night (*L*_night_), before energetically summing these levels with the daytime level *L*_day_ to obtain *L*_den_
N_60_	Number of events exceeding an A-weighted maximum sound level (*L*_AFmax_) of 60 dB per hour
RLS-19	“Richtlinien für den Lärmschutz an Straßen” (German guidelines for noise control along roads), national standardised method for road traffic noise modelling
SPL	Sound pressure level
WHO	World Health Organization

## References

[1] World Health Organization (2018). Environmental Noise Guidelines for the European Region.

[2] European Environment Agency (2026). Environmental Noise in Europe 2025.

[3] European Parliament, Council of the European Union (2002). Directive 2002/49/EC of the European Parliament and of the Council of 25 June 2002 relating to the assessment and management of environmental noise. Off. J. Eur. Communities.

[4] Guski R., Schreckenberg D., Schuemer R. (2017). WHO Environmental Noise Guidelines for the European Region: A Systematic Review on Environmental Noise and Annoyance. Int. J. Environ. Res. Public Health.

[5] Basner M., McGuire S. (2018). WHO Environmental Noise Guidelines for the European Region: A Systematic Review on Environmental Noise and Effects on Sleep. Int. J. Environ. Res. Public Health.

[6] Smith M.G., Cordoza M., Basner M. (2022). Environmental Noise and Effects on Sleep: An Update to the WHO Systematic Review and Meta-Analysis. Environ. Health Perspect..

[7] Lechner C., Schnaiter D., Siebert U., Böse-O’Reilly S. (2020). Effects of motorcycle noise on annoyance—A cross-sectional study in the Alps. Int. J. Environ. Res. Public Health.

[8] Schreckenberg D., Benz S., Kuhlmann J., Bilik J., Popp C., Heidebrunn F., Marki F. (2023). Motorcycle noise study Baden-Württemberg, part I: Long-term noise annoyance in residents living alongside busy motorcycle routes in the southwest of Germany. Proceedings of the 14th ICBEN Congress on Noise as a Public Health Problem, Belgrade, Serbia, 18–22 June 2023.

[9] Chang T.Y., Liu C.S., Bao B.Y., Li S.F., Chen T.I., Lin Y.J. (2011). Characterisation of road traffic noise exposure and prevalence of hypertension in central Taiwan. Sci. Total Environ..

[10] Samani O., Altinsoy M.E. (2021). Effects of frequency on annoyance caused by motorcycle noise. Fortschritte der Akustik—DAGA 2021.

[11] Paviotti M., Vogiatzis K. (2012). On the outdoor annoyance from scooter and motorbike noise in the urban environment. Sci. Total Environ..

[12] Czedik-Eysenberg I., Knauf D., Reuter C. Psychoakustische Aspekte der Lästigkeit von Motorradgeräuschen [Psycho-acoustic aspects of annoyance of motorcycle sounds]. Proceedings of the DGM 2015, Oldenburg, Germany.

[13] Huth C., Eberlei G., Liepert M. (2020). Überprüfung der Geräuschemissionen von Motorrädern im Realen Verkehr [Noise Emission of Motorcycles Under Real-Life Traffic Conditions].

[14] Guski R. (1999). Personal and social variables as co-determinants of noise annoyance. Noise Health.

[15] Miedema H.M.E., Vos H. (1999). Demografic and attitudinal factors that modify annoyance from transportation noise. J. Acoust. Soc. Am..

[16] Nelson P.M. (1985). Some Aspects of Motorcycle Noise and Annoyance.

[17] Lercher P., Sölder M. Exposure by motorbike noise in alpine residential areas—A case study in public health risk assessment. Proceedings of the Euronoise 2009, Edinburgh, Scotland.

[18] Benz S., Popp C., Heidebrunn F., Wack W., Schreckenberg D. (2023). Motorcycle noise study Baden-Württemberg, part II: Short-term noise annoyance in residents of busy motorcycle routes in the south of Germany. Proceedings of the 14th ICBEN Congress on Noise as a Public Health Problem, Belgrade, Serbia, 18–22 June 2023.

[19] Rachman R., Radjawane L.E. (2021). The effects of motorcycle proportion to traffic noise on residential area. Proceedings of the 4th International Conference on Sustainable Innovation 2020—Technology, Engineering and Agriculture (ICoSITEA 2020), Yogyakarta, Indonesia, 13–14 October 2020.

[20] Dendale P., Münzel T. (2025). Motorcycles as high-noise emitters, air polluters and cardiovascular threats: Time to regulate the risks. Eur. Heart J..

[21] Csikszentmihalyi M., Larson R. (1992). Validity and reliability of the experience-sampling method. J. Nerv. Ment. Dis..

[22] Föderation Deutscher Psychologenvereinigungen, Berufsverband Deutscher Psychologinnen und Psychologen, Deutsche Gesellschaft für Psychologie Berufsethische Richtlinien des Berufsverbandes Deutscher Psychologinnen und Psychologen e.V. und der Deutschen Gesellschaft für Psychologie e.V. zugleich Berufsordnung des Berufsverbandes Deutscher Psychologinnen und Psychologen e.V. (Fassung vom 14 September 2022). https://www.dgps.de/fileadmin/user_upload/PDF/Berufsetische_Richtlinien/BER-Foederation-20230426-Web-1.pdf.

[23] (2021). Acoustics—Assessment of Noise Annoyance by Means of Social and Socio-Acoustic Surveys.

[24] Conner Christensen T., Feldman Barrett L., Bliss-Moreau E., Lebo K., Kaschub C. (2003). A practical guide to experience-sampling procedures. J. Happiness Stud..

[25] Forschungsgesellschaft für Straßen- und Verkehrswesen (FGSV) (2019). Richtlinien für den Lärmschutz an Straßen (RLS 19), Ausgabe 2019.

[26] R Core Team (2022). R: A Language and Environment for Statistical Computing.

[27] Wickham H. (2016). ggplot2: Elegant Graphics for Data Analysis.

[28] Wickham H., François R., Henry L., Müller K. (2022). dplyr: A Grammar of Data Manipulation.

[29] Halekoh U., Højsgaard S., Yan J. (2006). The R package geepack for generalized estimating equations. J. Stat. Softw..

[30] Google NotebookLM. https://notebooklm.google/.

[31] Perplexity AI Perplexity. https://www.perplexity.ai/.

[32] Anthropic. Claude. https://www.anthropic.com/.

[33] Çorbacıoğlu Ş.K., Aksel G. (2023). Receiver operating characteristic curve analysis in diagnostic accuracy studies: A guide to interpreting the area under the curve value. Turk. J. Emerg. Med..

[34] Eidgenössische Kommission für Lärmbekämpfung (EKLB) (2021). Grenzwerte für Strassen-, Eisenbahn- und Fluglärm. Empfehlungen der Eidgenössischen Kommission für Lärmbekämpfung EKLB.

[35] Bartels S., Márki F., Müller U. (2015). The Influence of Acoustical and Non-Acoustical Factors on Short-Term Annoyance due to Aircraft Noise in the Field—The COSMA Study. Sci. Total Environ..

[36] Hoeger R. (2004). Aircraft Noise and Times of Day: Possibilities of Redistributing and Influencing Noise Exposure. Noise Health.

[37] Porter N.D., Kershaw A.D., Ollerhead J.B. (2000). Adverse Effects of Night-Time Aircraft Noise.

[38] Wang W., Ma H., Wang C. (2025). The Impact of Non-Acoustic Factors on Chinese Community Response to Noise: A Systematic Review. Int. J. Environ. Res. Public Health.

[39] Stallen P.J.M. (1999). A theoretical framework for environmental noise annoyance. Noise Health.

[40] Guski R., Felscher-Suhr U., Schuemer R. (1999). The Concept of Noise Annoyance: How International Experts See It. J. Sound Vib..

[41] Job R.S.F. (1999). Noise sensitivity as a factor influencing human reaction to noise. Noise Health.

[42] Lechner C., Lercher P. (2010). Action plan against motor bike noise—An Alpine case study. Proceedings of Internoise 2010, Lisbon, Portugal, 13–16 June 2010.

[43] Jäcker-Cüppers M. (2021). Motorradlärm—Aktuelle Konflikte, Positionen und Lösungsvorschläge [Motorcycle Noise—Current Conflicts, Positions, and Proposed Solutions]. Akust. J..

[44] Lechner C., Schnaiter D. (2021). Evaluierung der Maßnahmen zum Motorradlärm im Bezirk Reutte [Evaluation of Measures Against Motorcycle Noise in the Reutte District].

[45] Brink M., Schäffer B., Schreckenberg D. Key Aspects for Survey Data Processing and Statistical Analysis when Modelling Exposure-Annoyance Relationships. Proceedings of the Forum Acusticum Euronoise 2025.

[46] Shmueli G. (2010). To Explain or to Predict?. Stat. Sci..

[47] Lechner C., Schnaiter D. (2019). Motorradlärmstudie Außerfern 2019—Gesamtbericht [Motorcycle Study Aussenfern 2019—Main Report].

[48] Baliatsas C., van Kamp I., Swart W., Hooiveld M., Yzermans J. (2016). Noise sensitivity: Symptoms, health status, illness behavior and co-occurring environmental sensitivities. Environ. Res..

[49] Schaeffer B., Schalcher S., Brink M., Schreckenberg D. (2025). The association of residential greenery with noise annoyance depends on transport source and environmental context: Insights from the large-scale NORAH study. Environ. Res..

[50] Efron B., Tibshirani R.J. (1993). An Introduction to the Bootstrap.

[51] Benz S.L., Kuhlmann J., Bilik J., Liepert M., Schreckenberg D. (2025). Noise Annoyance and Sleep Disturbance Due to Road Traffic and Railway Noise in Germany. Int. J. Environ. Res. Public Health.

