# Supplementary material for: Motorcycle Noise Annoyance in Residential Areas Along Popular Leisure Routes
Source: Int J Environ Res Public Health. 2026 Jul 26;23(8):966. doi: 10.3390/ijerph23080966 (PMC13512848; doi:10.3390/ijerph23080966)
Supplement: Supplementary file 1 [file ijerph-23-00966-s001.zip › ijerph-4263081-supplementary.pdf]

Supplementary Materials

Motorcycle Noise Annoyance in Residential Areas Along Popular Leisure Routes

Dirk Schreckenberg [1,*], Sarah Leona Benz [2] , Julia Kuhlmann [1], Jonas Bilik [1], Christian Popp [3], Frank Heidebrunn [3], Wolfgang Wack [4], Ferenc Marki [5]

1 ZEUS GmbH, Centre for Applied Psychology, Environmental and Social Research, D-58093 Hagen, Germany; kuhlmann@zeusgmbh.de (J.K.)
2 D-44287 Dortmund, Germany
3 Lärmkontor GmbH, D-22767 Hamburg, Germany; c.popp@laermkontor.de (C.P.); f.heidebrunn@laermkontor.de (F.H.)
4 D-22941 Delingsdorf, Germany
5 Department of Networked Systems and Services, Faculty of Electrical Engineering and Informatics, Budapest University of Technology and Economics [MF1.1], H-1111 Budapest, Hungary; marki@vik.bme.hu
* Correspondence: schreckenberg@zeusgmbh.de

Academic Editor: Firstname Lastname

Received: date
Revised: date
Accepted: date
Published: date

Copyright: © 2026 by the authors. Submitted for possible open access publication under the terms and conditions of the Creative Commons Attribution (CC BY) license.

Table S1. Results of GEE logistic regressions for %HA on $L_{Aeq,1h}$

Parameters	B	SE	Wald	p
%HA motorcycle ~ $L_{Aeq,1h}$ (weekdays)				
Intercept	-5.546	0.634	76.4	< 0.001
$L_{Aeq,1h}$ motorcycle	0.072	0.012	34.05	< 0.001
alpha (correlation)	0.227	0.135		
Scale (Intercept)	1			
Modell-Fit:	AUC:			0.716
	McFadden Pseudo-R^2:			0.078
%HA passenger car ~ $L_{Aeq,1h}$ (weekdays)				
Intercept	-5.549	1.31	17.95	< 0.001
$L_{Aeq,1h}$ passenger car	0.064	0.025	6.76	0.009
alpha (correlation)	0.288	0.148		
Scale (Intercept)	1			
Modell-Fit:	AUC:			0.715
	McFadden Pseudo-R^2:			0.068
%HA lorry ~ $L_{Aeq,1h}$ (weekdays)				
Intercept	-6.05	0.663	83.25	< 0.001
$L_{Aeq,1h}$ lorry	0.071	0.013	29.62	< 0.001
alpha (correlation)	0.187	0.179		
Scale (Intercept)	1			
Modell-Fit:	AUC:			0.740
	McFadden Pseudo-R^2:			0.080
%HA motorcycle ~ $L_{Aeq,1h}$ (weekend)				
Intercept	-5.054	0.658	59.06	< 0.001
$L_{Aeq,1h}$ motorcycle	0.077	0.013	34.83	< 0.001
alpha (correlation)	0.472	0.128		
Scale (Intercept)	1			
Modell-Fit:	AUC:			0.671
	McFadden Pseudo-R^2:			0.054
%HA passenger car ~ $L_{Aeq,1h}$ (weekend)				
Intercept	-5.121	0.822	38.86	< 0.001
$L_{Aeq,1h}$ passenger car	0.058	0.016	14.22	< 0.001
alpha (correlation)	0.361	0.173		
Scale (Intercept)	1			
Modell-Fit:	AUC:			0.661
	McFadden Pseudo-R^2:			0.041

Table S2. Results of GEE logistic regressions for %HA on $L_{AFmax,1h}$

Parameters	B	SE	Wald	p
%HA motorcycle ~ $L_{AFmax,1h}$ (weekdays)				
Intercept	-6.007	0.943	40.59	0.000
$L_{AFmax,1h}$ motorcycle	0.052	0.012	17.52	0.000
alpha (correlation)	0.246	0.149		
Scale (Intercept)	1			
Modell-Fit:	AUC:			0.687
	McFadden Pseudo-R^2:			0.059
%HA passenger car ~ $L_{AFmax,1h}$ (weekdays)				
Intercept	-4.515	0.847	28.43	0.000
$L_{AFmax,1h}$ passenger car	0.034	0.012	8.23	0.004
alpha (correlation)	0.304	0.116		
Scale (Intercept)	1			
Modell-Fit:	AUC:			0.670
	McFadden Pseudo-R^2:			0.043
%HA lorry ~ $L_{AFmax,1h}$ (weekdays)				
Intercept	-6.767	1.03	43.18	0.000
$L_{AFmax,1h}$ lorry	0.057	0.014	16.97	0.000
alpha (correlation)	0.215	0.167		
Scale (Intercept)	1			
Modell-Fit:	AUC:			0.694
	McFadden Pseudo-R^2:			0.056
%HA motorcycle ~ $L_{AFmax,1h}$ (weekend)				
Intercept	-3.837	0.788	23.7	0.000
$L_{AFmax,1h}$ motorcycle	0.035	0.011	10.62	0.001
alpha (correlation)	0.476	0.096		
Scale (Intercept)	1			
Modell-Fit:	AUC:			0.612
	McFadden Pseudo-R^2:			0.026
%HA passenger car ~ $L_{AFmax,1h}$ (weekend)				
Intercept	-3.454	0.74	21.76	0.000
$L_{AFmax,1h}$ passenger car	0.020	0.011	3.47	0.063
alpha (correlation)	0.365	0.145		
Scale (Intercept)	1			
Modell-Fit:	AUC:			0.621
	McFadden Pseudo-R^2:			0.021

Table S3. Results of GEE logistic regressions for %HA on N_{60}

Parameters	B	SE	Wald	p
%HA motorcycle ~ N_{60} (weekdays)				
Intercept	-2.706	0.208	170.00	0.000
N_{60} motorcycle	0.050	0.008	38.98	0.000
alpha (correlation)	0.250	0.121		
Scale (Intercept)	1			
Modell-Fit:	AUC:			0.676
	McFadden Pseudo-R^2:			0.049
%HA passenger car ~ N_{60} (weekdays)				
Intercept	-2.271	0.190	143.26	0.000
N_{60} passenger car	0.002	0.001	2.96	0.085
alpha (correlation)	0.318	0.116		
Scale (Intercept)	1			
Modell-Fit:	AUC:			0.655
	McFadden Pseudo-R^2:			0.012
%HA lorry ~ N_{60} (weekdays)				
Intercept	-3.007	0.234	165.66	0.000
N60 lorry	0.033	0.010	10.13	0.001
alpha (correlation)	0.224	0.145		
Scale (Intercept)	1			
Modell-Fit:	AUC:			0.675
	McFadden Pseudo-R^2:			0.028
%HA motorcycle ~ N_{60} (weekend)				
Intercept	-1.634	0.157	108.11	0.000
N_{60} motorcycle	0.022	0.005	21.36	0.000
alpha (correlation)	0.472	0.088		
Scale (Intercept)	1			
Modell-Fit:	AUC:			0.610
	McFadden Pseudo-R^2:			0.021
%HA passenger car ~ N_{60} (weekend)				
Intercept	-2.292	0.187	150.68	0.000
N_{60} passenger car	0.003	0.001	9.50	0.002
alpha (correlation)	0.382	0.156		
Scale (Intercept)	1			
Modell-Fit:	AUC:			0.610
	McFadden Pseudo-R^2:			0.007

Table S4. Sensitivity analyses: Results of GEE logistic regressions for source-specific %HA with $L_{Aeq,1h,motorcycle}$, $L_{Aeq,1h,car}$ $L_{Aeq,1h,lorry}$ and the annoyance scores for noise of the respective other vehicle classes.

Model	Parameters	B	SE	Wald	p	VIF
	Outcome: %HA motorcycle					
M1a	Intercepte	-6.423	0.620	107.15	<0.001	--
	$L_{Aeq,1h\ motorcycle}$	0.099	0.012	66.70	<0.001	--
M1b	Intercept	-5.811	0.852	46.56	<0.001	
	$L_{Aeq,1h\ motorcycle}$	0.145	0.021	49.99	<0.001	4.76
	$L_{Aeq,1h\ car}$	-0.090	0.027	10.80	0.001	6.62
	$L_{Aeq,1h\ lorry}$	-0.020	0.009	4.84	0.028	2.29
	Annoyance passenger car	1.159	0.109	112.68	<0.001	1.32
	Annoyance lorry	-0.144	0.085	2.84	0.092	1.39
	n = 3101 ∣ clusters = 189 ∣ HA-events = 390 ∣ alpha = 0.175					
	Outcome: %HA passenger car					
	Intercept	-4.999	0.898	31.01	<0.001	--
	$L_{Aeq,1h\ car}$	0.055	0.017	10.64	0.001	--
	Intercept	-6.473	0.924	49.10	<0.001	
	$L_{Aeq,1h\ motorcycle}$	-0.008	0.017	0.23	0.631	3.75
	$L_{Aeq,1h\ car}$	0.051	0.024	4.61	0.032	5.17
	$L_{Aeq,1h\ lorry}$	-0.014	0.008	3.19	0.074	2.38
	Annoyance passenger lorry	0.554	0.082	45.18	<0.001	1.19
	Annoyance motorcycle	0.678	0.089	57.89	<0.001	1.20
	n = 3101 ∣ clusters = 189 ∣ HA-events = 294 ∣ alpha = 0.260					
	Outcome: %HA lorry					
	Intercept	-6.190	0.832	55.37	<0.001	--
	$L_{Aeq,1h\ car}$	0.071	0.017	17.06	<0.001	--
	Intercept	-10.156	1.611	39.76	<0.001	
	$L_{Aeq,1h\ motorcycle}$	-0.054	0.020	7.35	0.007	3.19
	$L_{Aeq,1h\ car}$	0.041	0.055	0.54	0.463	4.82
	$L_{Aeq,1h\ lorry}$	0.071	0.036	3.95	0.047	2.50
	Annoyance passenger car	1.478	0.159	86.40	<0.001	1.37
	Annoyance motorcycle	-0.024	0.129	0.03	0.852	1.58
	n = 3101 ∣ clusters = 189 ∣ HA-events = 100 ∣ alpha = 0.058					

Table S5. Sensitivity analyses: Results of GEE logistic regressions for source-specific %HA with $L_{Aeq,1h,motorcycle}$, $L_{Aeq,1h,car}$, $L_{Aeq,1h,lorry}$ and the annoyance scores for noise of the respective other vehicle classes (weekdays)

Model	Parameters	B	SE	Wald	p	VIF
	Outcome: %HA motorcycle					
M1a	Intercept	-5.685	0.667	72.70	<0.001	--
	$L_{Aeq,1h\ motorcycle}$	0.074	0.013	32.92	<0.001	--
M1b	Intercept	-6.034	1.012	35.57	<0.001	
	$L_{Aeq,1h\ motorcycle}$	0.102	0.025	16.79	<0.001	3.48
	$L_{Aeq,1h\ car}$	-0.060	0.034	3.10	0.078	5.87
	$L_{Aeq,1h\ lorry}$	-0.018	0.014	1.72	0.189	2.96
	Annoyance passenger car	1.098	0.135	66.01	<0.001	1.67
	Annoyance lorry	0.079	0.126	0.39	0.533	1.70
	n = 1854 ǀ clusters = 185 ǀ HA-events = 138 ǀ alpha = 0.136					
	Outcome: %HA passenger car					
	Intercept	-5.818	1.152	25.51	<0.001	--
	$L_{Aeq,1h\ car}$	0.069	0.022	10.33	0.001	--
	Intercept	-6.926	1.089	40.48	<0.001	
	$L_{Aeq,1h\ motorcycle}$	0.004	0.019	0.05	0.815	3.59
	$L_{Aeq,1h\ car}$	0.053	0.029	3.39	0.066	5.82
	$L_{Aeq,1h\ lorry}$	-0.025	0.016	2.23	0.136	2.95
	Annoyance passenger lorry	0.791	0.139	32.27	<0.001	1.37
	Annoyance motorcycle	0.552	0.122	20.36	<0.001	1.30
	n = 1854 ǀ clusters = 185 ǀ HA-events = 164 ǀ alpha = 0.182					
	Outcome: %HA lorry					
	Intercept	-6.006	0.686	76.73	<0.001	--
	$L_{Aeq,1h\ car}$	0.070	0.014	26.62	<0.001	--
	Intercept	-9.955	1.746	32.49	<0.001	
	$L_{Aeq,1h\ motorcycle}$	-0.034	0.022	2.45	0.117	3.60
	$L_{Aeq,1h\ car}$	0.016	0.047	0.12	0.734	5.91
	$L_{Aeq,1h\ lorry}$	0.070	0.028	6.53	**0.011**	2.93
	Annoyance passenger car	1.456	0.194	56.02	<0.001	1.68
	Annoyance motorcycle	0.176	0.137	1.64	0.200	1.63
	n = 1854 ǀ clusters = 185 ǀ HA-events = 82 ǀ alpha = 0.077					

Table S6. Sensitivity analyses: Results of GEE logistic regressions for source-specific %HA with $L_{\mathrm{Aeq,1h\ ,motorcycle}}$, $L_{\mathrm{Aeq,1h,car}}$ $L_{\mathrm{Aeq,1h,lorry}}$ and the annoyance scores for noise of the respective other vehicle classes (weekend)

Model	Parameters	B	SE	Wald	p	VIF
Outcome: %HA motorcycle						
M1a	Intercept	-5.620	0.754	55.57	<0.001	--
	$L_{\mathrm{Aeq,1h\ motorcycle}}$	0.089	0.015	35.92	<0.001	--
M1b	Intercept	-6.311	1.000	39.81	<0.001	
	$L_{\mathrm{Aeq,1h\ motorcycle}}$	0.142	0.026	30.52	<0.001	4.32
	$L_{\mathrm{Aeq,1h\ car}}$	-0.102	0.033	9.48	0.002	5.32
	$L_{\mathrm{Aeq,1h\ lorry}}$	0.014	0.012	1.31	0.252	2.22
	Annoyance passenger car	1.155	0.141	66.67	<0.001	1.25
	Annoyance lorry	0.043	0.130	0.11	0.741	1.22
n = 1247 ǀ clusters = 179 ǀ HA-events = 252 ǀ alpha = 0.305						
Outcome: %HA passenger car						
	Intercept	-5.491	0.901	37.11	<0.001	--
	$L_{\mathrm{Aeq,1h\ car}}$	0.067	0.017	15.93	<0.001	--
	Intercept	-7.354	1.156	40.47	<0.001	
	$L_{\mathrm{Aeq,1h\ motorcycle}}$	-0.055	0.028	3.91	0.048	4.70
	$L_{\mathrm{Aeq,1h\ car}}$	0.087	0.034	6.76	**0.009**	5.43
	$L_{\mathrm{Aeq,1h\ lorry}}$	-0.002	0.012	0.04	0.848	2.21
	Annoyance passenger lorry	0.440	0.165	7.10	0.008	1.11
	Annoyance motorcycle	0.999	0.133	56.74	<0.001	1.22
n = 1247 ǀ clusters = 179 ǀ HA-events = 130 ǀ alpha = 0.329						

Table S7. Cluster bootstrap of the source-specific exposure-response coefficients (hourly $L_{\mathrm{Aeq,1h}}$), MotoApp study, total period (weekdays and weekends)

Outcome (%HA)	Parameters	B	Bias	BCa 95% CI lower	BCa 95% CI upper	n (clusters)
Motorcycle	$L_{\mathrm{Aeq,1h\ motorcycle}}$	0,093	0,001	0,070	0,116	3,610 (191)
Passenger car	$L_{\mathrm{Aeq,1h,\ passenger\ car}}$	0,047	0,002	-0,001	0,083	3,622 (191)
Lorry	$L_{\mathrm{Aeq,1h,lorry}}$	0,071	0,002	0,027	0,105	3,115 (189)

Note. Cluster bootstrap resampling whole participants (with replacement, 5,000 replications, seed = 2026); each draw received a new unique cluster ID. B is the LAeq,1h slope of the source-specific GEE logistic model (%HA ~ LAeq,1h, exchangeable working correlation). Bias = mean bootstrap estimate minus original estimate. BCa = bias-corrected and accelerated 95% interval. A BCa interval excluding zero corresponds to significance at the 5% level. The bootstrap was run on the pooled (weekday + weekend) sample as a robustness check of the exposure-response relationship; it reflects internal stability to participant composition and does not address selection of survey non-respondents.
